# The Schultz MIDI Benchmarking Toolbox for MIDI interfaces, percussion pads, and sound cards

**DOI:** 10.3758/s13428-018-1042-7

**Published:** 2018-04-17

**Authors:** Benjamin G. Schultz

**Affiliations:** Basic & Applied Neurodynamics Laboratory, Department of Neuropsychology & Psychopharmolcology, Maastricht University, Universiteitssingel 40, 6229 ER Maastricht, The Netherlands

**Keywords:** Musical Instrument Digital Interface (MIDI), Auditory feedback, Sensorimotor synchronization, Motor timing, Microcontrollers

## Abstract

The Musical Instrument Digital Interface (MIDI) was readily adopted for auditory sensorimotor synchronization experiments. These experiments typically use MIDI percussion pads to collect responses, a MIDI–USB converter (or MIDI–PCI interface) to record responses on a PC and manipulate feedback, and an external MIDI sound module to generate auditory feedback. Previous studies have suggested that auditory feedback latencies can be introduced by these devices. The Schultz MIDI Benchmarking Toolbox (SMIDIBT) is an open-source, Arduino-based package designed to measure the point-to-point latencies incurred by several devices used in the generation of response-triggered auditory feedback. Experiment 1 showed that MIDI messages are sent and received within 1 ms (on average) in the absence of any external MIDI device. Latencies decreased when the baud rate increased above the MIDI protocol default (31,250 bps). Experiment 2 benchmarked the latencies introduced by different MIDI–USB and MIDI–PCI interfaces. MIDI–PCI was superior to MIDI–USB, primarily because MIDI–USB is subject to USB polling. Experiment 3 tested three MIDI percussion pads. Both the audio and MIDI message latencies were significantly greater than 1 ms for all devices, and there were significant differences between percussion pads and instrument patches. Experiment 4 benchmarked four MIDI sound modules. Audio latencies were significantly greater than 1 ms, and there were significant differences between sound modules and instrument patches. These experiments suggest that millisecond accuracy might not be achievable with MIDI devices. The SMIDIBT can be used to benchmark a range of MIDI devices, thus allowing researchers to make informed decisions when choosing testing materials and to arrive at an acceptable latency at their discretion.

**Electronic supplementary material:**

The online version of this article (10.3758/s13428-018-1042-7) contains supplementary material, which is available to authorized users.

The Musical Instrument Digital Interface (MIDI) is a digital communication protocol for electronic musical instruments, computers, and audio devices that allows them to communicate between one another to produce music and sound effects and to record responses. MIDI is, arguably, the most widely used technical specification for digital musical instruments, and it was popular even before it was first published (International MIDI Association, 1983). Besides being used by performers from Prince to present-day bands like Snarky Puppy, MIDI instruments have commonly been used in sensorimotor synchronization experiments to measure the timing of actions and to generate auditory feedback (cf. Repp. 2005; Repp & Su, 2013). In these experiments, participants aim to synchronize their responses to an external stimulus (e.g., a metronome or auditory sequence), and the timing of responses relative to events (or beats) is recorded. Despite the wide use and success of MIDI, the temporal latencies of the recorded response timings and auditory feedback have rarely been benchmarked (but see Mills, van der Steen, Schultz, & Keller, 2015; Repp, 2010; Schultz & van Vugt, 2016) and are rarely specified by the manufacturer. The aim of the present study was to describe tools to benchmark the latencies of several commercially available MIDI devices—namely, the Schultz MIDI Benchmarking Toolbox (SMIDIBT). The SMIDIBT affords the opportunity for researchers to measure the timing error within MIDI devices (and configurations thereof), in order to make informed decisions regarding the choice of experimental apparatus, sample sizes (and statistical power, given said error), and interpretation of the results (e.g., negative mean asynchrony; see Repp, 2005).

Previous sensorimotor synchronization experiments have used various combinations of MIDI response devices (e.g., pianos or percussion pads), MIDI-to-PC conversion interfaces (e.g., MIDI–USB converters), and external MIDI sound modules to produce auditory feedback (see Table 1 and Fig. 1a). Similarly, the computer software used to record responses and control auditory feedback, such as FTAP (Finney, 2001) or Max-MSP (Cycling’74, 2014), differs between studies. Although it would be difficult to benchmark some of these setups for various reasons (e.g., discontinued stock, financial expense, custom designs/scripts, PC specifications), it is possible to provide a benchmarking toolbox so researchers can test their own experimental setups. Here I present the SMIDIBT for MIDI devices and test some of the most frequently used devices or the newer (or similar) versions (see Table 1). Since Schultz and van Vugt (2016) claimed that one of the advantages of an open-source microcontroller response device (other than sub-millisecond auditory feedback) is that it is more affordable than MIDI-based setups, in the present study I also examined the performance of several affordable devices—namely, an Alesis PercPad ($199 US), a LogiLink MIDI–USB cable ($20 US), and a MIDItech Pianobox ($90 US). I start by describing the hardware and software of the SMIDIBT and exploring the limits of MIDI-based communication (Exp. 1). I then investigate some of the assumptions surrounding the use of the MIDI protocols: specifically, USB polling rates, the asserted 1-ms resolution of MIDI devices (e.g., Large, Fink, & Kelso, 2002; Ravignani, Delgado, & Kirby, 2016), and whether MIDI messages and audio output are synchronous (see Maidhof, Kästner, & Makkonen, 2014). The present study compared the latencies of the various MIDI devices to the critical value of 1 ms, since this is the latency assumed (and, perhaps, desired) in some experiments (e.g., Large et al., 2002; Ravignani et al., 2016). The primary aim is to understand the hardware and software limitations that may influence latencies when using individual MIDI devices or chaining several MIDI devices, and show users how to measure these latencies themselves. To improve accessibility, I have also included a step-by-step guide in the supplementary materials (see the SMIDIBT Reference Manual) that contains less technical descriptions aimed at novices who perform auditory feedback experiments.

**Table 1:** List of the commercially available devices used in previous experiments and/or tested in the present study (demarcated with a *)

Type	Device	References
MIDI–USB interface	LogiLink MIDI-USB^*^	N/A
	M-Audio UNO^*^	Mills et al. (2015)
	MIDI Man (MIDI Sport)	Collyer et al. (1997); Schultz & van Vugt (2016)
	Roland UM-ONE^*^	Rogers et al. (2014)
MIDI–PCI interface	Labway Soundboard D66^*^	N/A
	Sound Blaster Live PCI^*^	Pecenka & Keller (2011); Pfordresher & Benitez (2007); Pfordresher & Dalla Bella (2011); Pfordresher & Kulpa (2011)
	TerraTec TT Solo 1-NL^*^	N/A
Percussion pad	Alesis PercPad^*^	Sadakata et al. (2008)
	Roland Handsonic 20^*^ (Roland Handsonic 10, 15)	Hurley, Martens, & Janata (2014); Janata, Tomic, & Haberman (2012); Mills et al. (2015); Pecenka & Keller (2011); Schultz & van Vugt (2016)
	Roland SPD6^*^	London et al. (2009); Pfordresher & Benitez (2007); Pfordresher & Dalla Bella (2011); Pfordresher & Kulpa (2011); Repp (2010); Repp & Knoblich (2007); Repp, London, & Keller (2005, 2012)
MIDI sound modules	MIDItech Pianobox^*^	N/A
	Roland Mobile Studio Canvas SD-50^*^ (Edirol StudioCanvas SD-80)	Pfordresher & Benitez (2007); Pfordresher & Dalla Bella (2011); Pfordresher & Kulpa (2011)
	Yamaha TX81Z	Schultz & van Vugt (2016)
MIDI piano sound modules	Kawai CL25^*^	N/A
	Korg MicroX^*^	N/A
	Roland RD-250s	Repp (2010); Repp & Marcus (2010)
	Yamaha Clavinova CLP-150	Kaiser & Keller (2011); Pecenka & Keller (2011); Repp & Keller (2010); Repp, London, & Keller (2012)

**Fig. 1:**
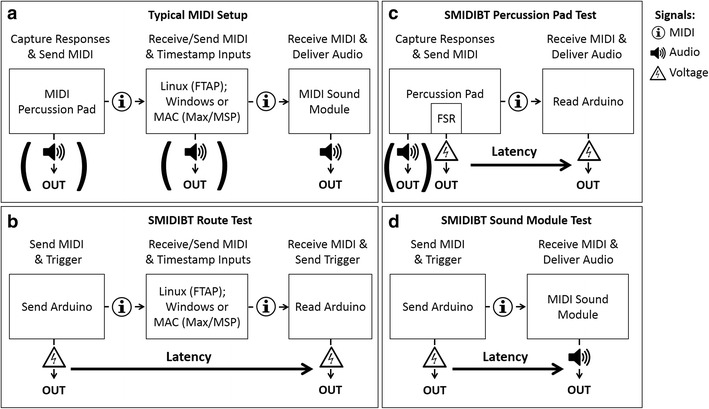
Layouts of a typical MIDI setup (a) and of the SMIDIBT setups that can be used to test the latencies of MIDI messages that are sent through computers and MIDI serial devices (b), the latencies of MIDI and audio from percussion pads (c), and the latencies of audio generated by MIDI sound modules (d). Each of the three components that can be sources of latencies in a typical MIDI setup is represented in panels b (middle section of a typical MIDI setup), c (first section of a typical MIDI setup), and d (final section of a typical MIDI setup). The OUT audio and voltages can be compared in to her measure the veridical timing between inputs and outputs

## MIDI-to-USB conversion and USB polling

The speed at which MIDI messages can be sent and read by the USB port and other MIDI devices is determined by the number of bits-per-second (bps), called the *baud rate* (or serial transfer rate). For MIDI, this transfer rate is set to 31,250 bps, and each MIDI message is three bytes. At this rate, Kieley (1991) suggests that the temporal resolution of the MIDI system is approximately 1 ms. Collyer, Boatright-Horowitz, and Hooper (1997) corroborate this resolution, reporting a mean MIDI message resolution of 1.04 ms (*SD* = 0.02 ms). However, this resolution might be affected when MIDI messages are sent and received by different MIDI interfaces, such as MIDI–USB interfaces. USB human interface devices (HID) poll to receive bytes from external devices (Garney, 1996; Schultz & van Vugt, 2016); every few milliseconds the serial port is sampled and, if new information is available, the data in the buffer are read. The default polling rate is about 8 ms between reads on most systems and some devices come with dedicated drivers to achieve lower polling rates. The MIDI–USB specification is stated to transfer MIDI messages using bulk endpoints, whereby the information does not poll, per se, but is only sent or read when unallocated bandwidth on the bus is available (Garney, 1996; Universal Serial Bus Specification, 2000). Bulk transfers are generally used for time-insensitive communication, because there is no guarantee of a minimum or consistent latency (Peacock, 2010). There has been some disagreement regarding whether bulk transfer communication avoids the delays introduced by polling or, instead, introduces different or more variable delays (Finney, 2016). In Experiment 2 I benchmarked the latency introduced by MIDI–USB and conventional Peripheral Component Interconnect (PCI) sound card interfaces, the latter requiring some basic expertise in computer construction to install a PCI card inside a computer case. I further tested the hypothesis that MIDI–USB interfaces poll by comparing three MIDI–USB devices with three PCI devices that do not rely on MIDI-to-USB conversion.

## MIDI percussion pads

MIDI percussion pads can be benchmarked in two ways. First, the latency of the audio produced by the device itself can be measured, as some experiments have presented feedback directly from the device (e.g., Ravignani et al., 2016). Second, the latency of the MIDI message can be measured to benchmark the delay and variability of the recorded response relative to the actual response. To the knowledge of the author, only two studies have benchmarked the performance of MIDI percussion pads in isolation from MIDI–USB/MIDI–PCI devices and/or MIDI sound modules (but see Repp, 2010, for end-to-end latencies). Mills et al. (2015) reported a response delay of approximately 5 ms for a MIDI response from a Roland Handsonic 10, but the method used to benchmark the device was not described. Schultz and van Vugt (2016) found that the Roland Handsonic 15 produced auditory feedback with significantly larger latencies than two Arduino-based setups, but they did not report the latency of the MIDI message itself. However, only two MIDI percussion pads were tested in these two experiments, and both belonged to the Roland Handsonic series. It is possible that other percussion pads may have different latencies or greater sensitivity to veridical responses. Here, three commercially available MIDI percussion pads were tested: the Alesis Percussion Pad (PercPad), the Roland Sampling Percussion Drumpad 6 (SPD6), and the Roland Handsonic Hand Percussion Drumpad 20 (HPD20). The SMIDIBT measured latencies using a force-sensitive resistor (FSR) placed on the percussion pad. Three latencies were measured: (1) between the FSR being pressed and the device’s audio output, (2) between the FSR being pressed and the reception of the MIDI message, and (3) between the audio output and the reception of the MIDI message. The latter was examined to assess whether the audio onset time is synchronous with the MIDI message (see Maidhof et al., 2014).

## MIDI sound modules

When auditory feedback is presented to participants in motor experiments that use MIDI, the most common method is to use a MIDI sound module. MIDI sound modules receive MIDI messages that trigger audio through the use of MIDI sound banks or, alternatively, a bank of patches (e.g., audio files of .wav or .mp3 extensions) associated with an instrument or note. MIDI sound banks come in several forms: The standard General MIDI specification (GM), a sound bank of 128 musical instruments (MIDI Manufacturers Association, 1996) and extended sound banks, such as Roland’s General Standard (GS), Yamaha’s Extended MIDI (XG), and General MIDI 2 (GM2). Various models of MIDI sound modules exist, each with their own sound banks that conform to the MIDI standard. The MIDI specification (e.g., GM, GS, XG, or GM2) used in MIDI sound modules is often specified by the manufacturer but might not produce similar auditory feedback latencies between devices that use the same sound module and sound bank. Moreover, the latencies for audio output once the MIDI message is received is not specified by the manufacturer and will vary between each sound on the basis of the temporal envelope. The latency of MIDI sound module audio is difficult to test unless one can accurately measure the time the MIDI message was sent or can split the MIDI message and compare the arrival of the MIDI message with the audio onset. The latter solution may produce additional delays as the splitters might read the MIDI message and resend the duplicated messages to two MIDI outs or, alternatively, amplify the MIDI output when splitting the output. To solve this problem, the SMIDIBT discussed herein can acquire consistent and reliable MIDI sound module audio latencies by using an Arduino microcontroller.

For practical reasons, in the present study I tested a selection of sound modules, including some that have been used in sensorimotor synchronization experiments (see Table 1). Two dedicated MIDI sound modules were tested: the one that is most used in these experiments (Roland Mobile Studio Canvas SD-50, or similar) and one that is less expensive (MIDItech Pianobox). It should be noted that some MIDI instruments contain an internal sound module that can be used to deliver auditory feedback (see Pecenka & Keller, 2011; Repp, 2010; Repp & Knoblich, 2007; Repp et al., 2005, 2012; Pfordresher & Benitez, 2007; Pfordresher & Dalla Bella, 2011; Pfordresher & Kulpa, 2011). Therefore, two MIDI keyboards were also tested—namely, a Kawai CL25 and a Korg MicroX synthesizer. Given that the MIDI protocol is a consistent standard, latencies were not expected to differ between devices that used the same (or similarly named) sound banks within the same specification.

## The Schultz MIDI Benchmarking Toolbox

The SMIDIBT contains schematics and code (Arduino IDE and MATLAB) to facilitate the accurate benchmarking of MIDI sound modules (and other MIDI devices) using an Arduino MIDI setup in conjunction with any audio sound card (i.e., digital mixer), analog input box, or oscilloscope. These tools are designed to allow experimenters to assess the audio latencies of their device and sound bank(s) prior to commencing experiments. Because it is not possible to comprehensively test the latencies of every available configuration, the present experiments focused on a selection of devices and sound banks that share similar sound bank names and, also, the sounds that demonstrate the lowest and highest latencies for each device. I provide examples of how the SMIDIBT can be used, and present a subset of results from my own tests using an informed selection of devices. Since it is near impossible to test every MIDI device available, the SMIDIBT and associated scripts used in this experiment are freely available so that other researchers can benchmark their own MIDI devices and report the latency of their chosen apparatus and stimuli within their articles.

The present study examined several different components of a typical MIDI-based experimental setup. Experiment 1 measured the durations of several steps of MIDI message transmission including the duration of sending a message, the duration for a sent message to be received, the duration of reading a message, and the total duration. Experiment 1 further served to validate the SMIDIBT as a tool to attain sub-millisecond timestamps for MIDI devices. In Experiment 2 I benchmarked the latencies introduced by different MIDI interface devices—specifically, three MIDI–USB devices and three PCI sound cards. This was conducted using the SMIDIBT route test and the FTAP loop test (Finney, 2001). Experiment 3 was designed to benchmark the audio and MIDI latencies of three MIDI percussion pads using the SMIDIBT. Finally, in Experiment 4 I assessed the auditory feedback latency of MIDI sound modules and sound banks using the SMIDIBT.

## Experiment 1: Benchmarking tools

Experiment 1 benchmarked how long it takes an Arduino to send, receive, and read MIDI messages in the SMIDIBT. I further explored the possibility that the speed of MIDI message processing is bottlenecked by the baud rate, by testing four different baud rates: the MIDI-specified rate of 31,250 bps, 50% of this rate (15,625 bps), 150% of this rate (46,875 bps), and 200% of this rate (62,500 bps). Moreover, I tested two different software-based methods for receiving MIDI messages to compare how different methods of parsing MIDI messages may affect the latency. These tests serve as references for how other MIDI devices might process MIDI messages depending on different specifications. Because suppliers do not provide details of the hardware or software used to process MIDI messages, these tests simply show how the Arduino can be used to send and receive MIDI messages and approximate the delays for other devices. To approximate differences between different printed circuit board (PCB) architectures and central processing units (CPUs), I also tested two different Arduinos, namely, the Arduino Mega (16 MHz) and the Arduino Due (84 MHz).

### SMIDIBT hardware and software

**Fig. 2:**
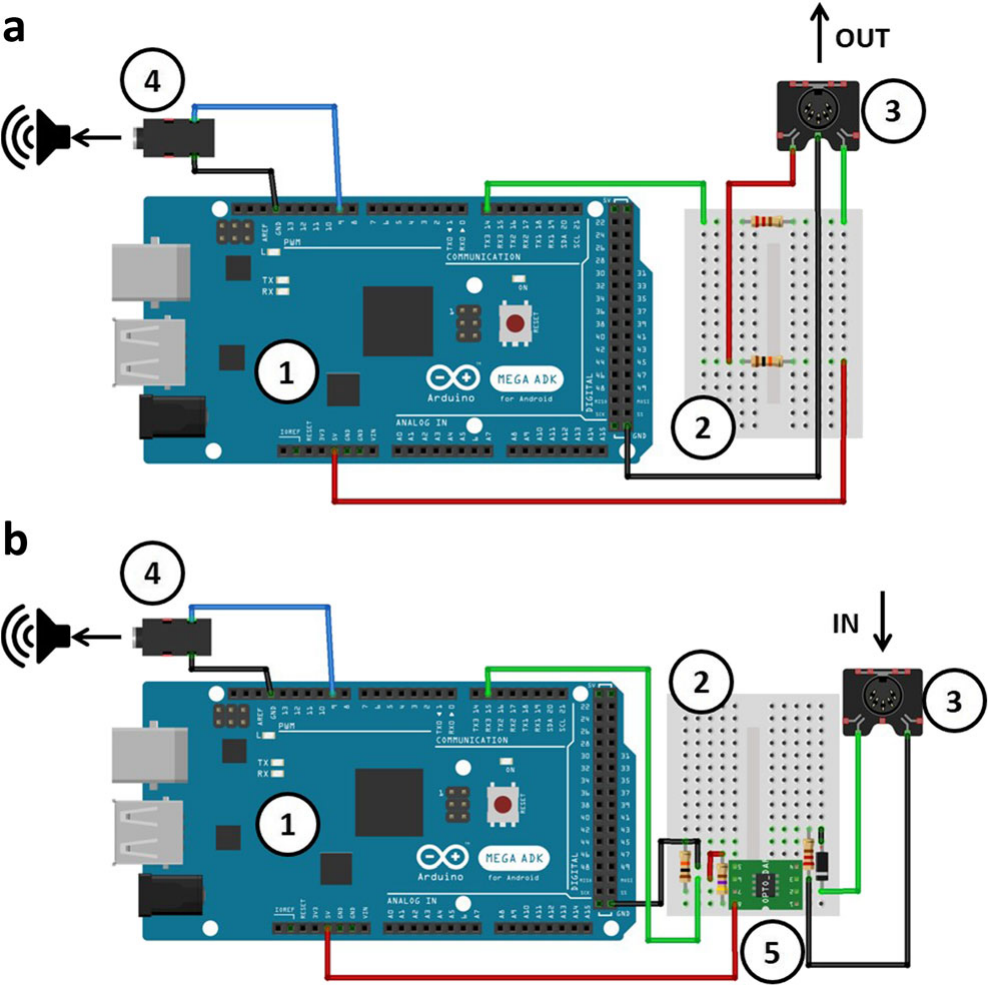
Schematic wiring diagrams for the Schultz MIDI Benchmarking Toolbox setup. The component numbers correspond to (1) Arduino Mega, (2) Breadboard, (3) MIDI connector, (4) audio jack, and (5) an optocoupler (6N138). Electric wires are indicated by black (grounds), red (power), green (MIDI signals), and blue (audio signals) wires. The top panel (a) shows the “send Arduino.” The green wire is connected to a 220-Ω resistor, and the red wire is connected to a 10-kΩ resistor. If using an Arduino Due, connect the red wire to the 3.3-V pin instead of the 5-V pin. The bottom panel (b) shows the “read Arduino.” The resistors from left to right are 10 kΩ, 470 Ω, and 220 Ω. Note the direction of the black diode (1N4148) on the far right, as it is unidirectional. This wiring diagram will allow prospective users to precisely reproduce the setup from the hardware components. This figure was created using fritzing (Knörig, Wettach, & Cohen, 2009)

The SMIDIBT requires up to two Arduino units, each performing a different task. The “send Arduino” sends the MIDI messages (see Fig. 2a), and the “read Arduino” receives MIDI messages (see Fig. 2b). I used the Arduino Mega and Arduino Due because both of these units have more than one serial communication port—that is, more than one pair of transmit (TX) and receive (RX) pins. This allows USB communication to and from the Arduino that is independent from the MIDI communication. The Arduino sent and received MIDI messages through a five-pin MIDI connector (CUI Inc.) that either sent MIDI messages from pin 14 (TX3; Fig. 2a) or received MIDI messages from pin 15 (RX3; Fig. 2b). Duty cycle changes were sent from PWM pin 9 via a separate headphone jack for each Arduino. The code used to benchmark the send durations and read durations sends analog triggers by changing the duty cycle of a PWM pin at the beginning and end of the read messages (see Appendix 1). For sending (send Arduino), the duty cycle changed from low to high just before the three-byte MIDI message was sent, and returned to low once the third byte had been sent. For reading (read Arduino), the duty changed from low to high when the first byte was identified as a MIDI command (144 = note onset, 153 = drum onset, 128 = note offset) and returned to low once the third byte had been read. These duty cycle changes can then be read by any apparatus that can record voltages, such as an audio sound card (i.e., digital mixer) or an oscilloscope. Onsets and offsets (i.e., MIDI triggers) can then be extracted through signal-processing techniques—in this case, custom-made MATLAB scripts that are available at the Basic and Applied NeuroDynamics laboratory website (https://band-lab.com\smidib_toolbox). Furthermore, these triggers can be used to measure the temporal delays of throughput MIDI devices (e.g., MIDI–USB converters; see Fig. 1b and Exp. 2), response devices (e.g., MIDI percussion pads; see Fig. 1c and Exp. 3), and auditory feedback devices (e.g., MIDI sound modules; see Fig. 1d and Exp. 4). Experiment 1 tested the latencies of sending and receiving MIDI messages using the SMIDIBT in the absence of these devices, to gain a better understanding of the MIDI protocol and ensure that the SMIDIBT can benchmark with sub-millisecond precision.

### Design and hypotheses

The dependent variables were the duration of sending a MIDI message (send duration), the duration between the message being sent and initially received (transit duration), the duration of receiving and reading a MIDI message (read duration), and the total duration from the time just prior to sending the message until the time at which the message has been read (total duration). The independent variables were the baud rate (four levels: 15,625, 31,250, 46,875, 62,500 bps), PCB (two levels: Mega, Due), and read method (two levels: single byte, all bytes). For the read method, the “single byte” method read each byte as it arrived, and the “all bytes” method waited for all three bytes of a MIDI message before a byte was read (see the code in Appendix 1). I hypothesized that the “single-byte” method would produce shorter total durations than the “all-bytes” method, due to the waiting time for all three bytes in the latter. I further hypothesized that all durations would decrease as the baud rate increased. Finally, I hypothesized that the total duration of sending and reading MIDI messages would occur within 1 ms.

### Method

#### Materials

Audio data were collected on an Intel Xeon E5-1650 PC (3.5 GHz, 32 GB RAM) running Windows 7. Reaper audio software recorded duty cycle changes (low, high) from the two Arduinos of the same model (either Arduino Mega or Arduino Due), and these signals were recorded by an M-Audio M-Track mixer at a sampling rate of 44100 Hz, allowing a resolution of 0.023 ms.

#### Procedure

A “note on” MIDI message was sent every 10 ms, and a “note off” MIDI message was sent 5 ms after the “note on” message, thus alternating between “note on” and “note off” every 5 ms. This alternation was performed 4,002 times in each trial, for 20 trials per condition. For reading in the “single-byte” method, the duty cycle changed from low to high when the first byte was identified as a MIDI command (144 = note onset, 153 = drum onset, 128 = note offset) and returned to low once the third byte had been read. For reading in the “all-bytes” method, the duty cycle changed from low to high once all three bytes were available and the first byte was identified as a MIDI command, and it returned to low once the three bytes had been read.

#### Onset and offset extraction

The trigger onsets (changes from low to high) were estimated by the first moment that the normalized voltage (ranging from – 1 to 1) surpassed a threshold of 0.2 and then pinpointed by finding the point at which the previous sample (*n* – 1) no longer had a lower voltage. The trigger offsets (changes from high to low) were estimated as the first moment that the normalized voltage fell below 0.015 and then pinpointed by finding the point at which the previous sample (*n* – 1) no longer had a higher voltage. Send durations were calculated as the difference between the trigger offset and the trigger onset for the “send Arduino.” Transit durations were calculated as the difference between the trigger onset of the “read Arduino” and the trigger offset of the “send Arduino.” Read durations were calculated as the difference between the trigger offset and trigger onset of the “read Arduino.” Total durations were calculated as the difference between the trigger offset of the “read Arduino” and the trigger onset of the “send Arduino.”

### Results

#### Statistical analysis

Separate repeated measures analyses of variance (ANOVAs) were conducted for the dependent variables send duration, transit duration, read duration, and total duration. The within-subjects factors were baud rate (15,625, 31,250, 46,875, 62,500 bps), method (single byte, all bytes), and PCB (Arduino Mega, Arduino Due). ANOVAs were performed using the ezANOVA function of the ez library (Lawrence, 2015) for the R package of statistical computing (R Core Team, 2013). *F* statistics, significance values, and effect sizes (generalized eta squared; *η*_G_^2^) are reported. Pair-wise contrasts were computed using generalized linear hypothesis testing for Tukey contrasts, using the glht function in the multcomp library (Hothorn, Bretz, & Westfall, 2008). The means, standard deviations, and value ranges are provided in Appendix 2.

#### Duration analyses

**Fig. 3:**
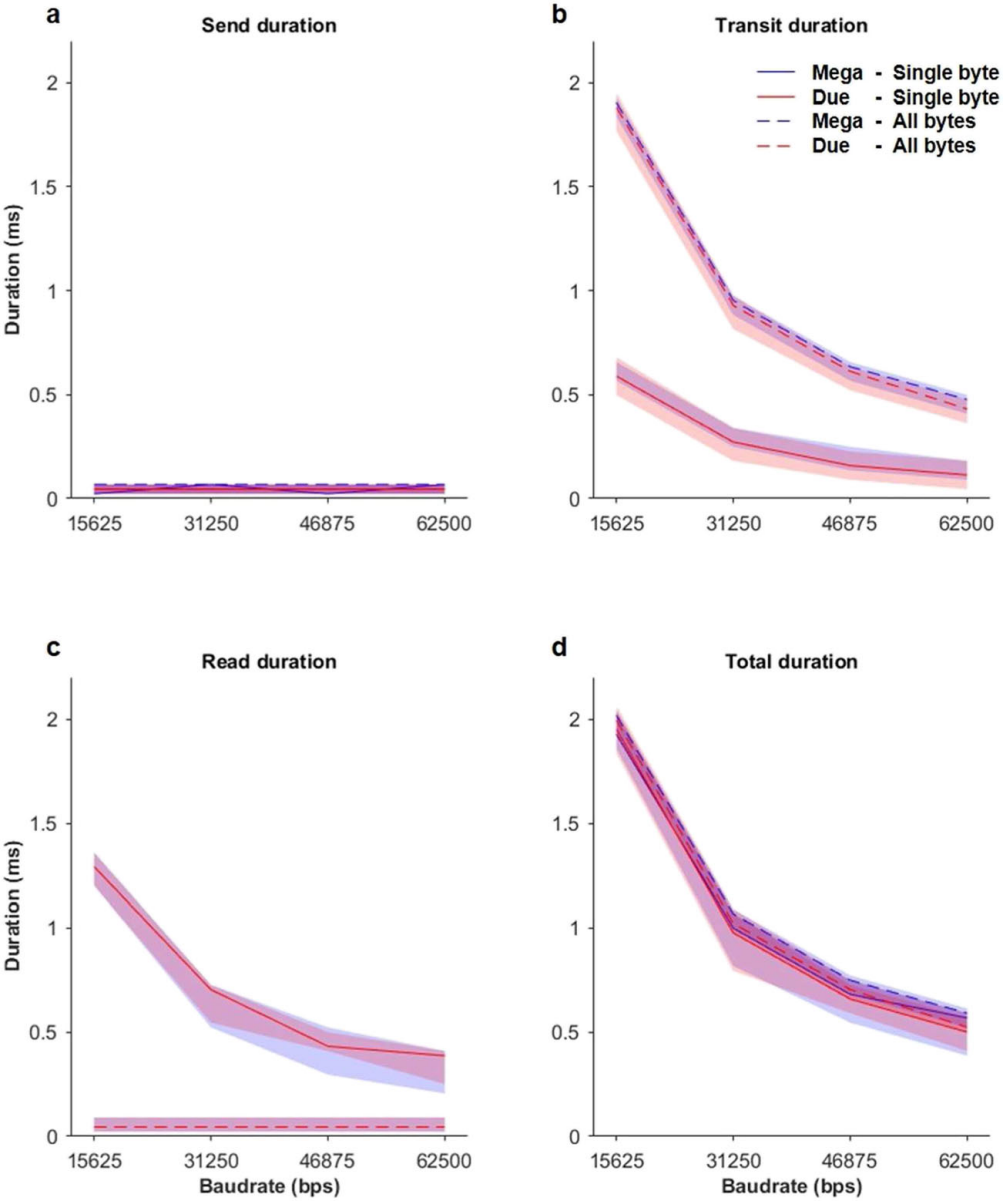
Mean (lines) and range (shaded regions) for send duration (a), transit duration (b), read duration (c), and total duration (d) for the Arduino Mega (blue) and Arduino Due (red) using the “single-byte” method (solid lines) and the “all-bytes” method (dashed lines) for all four baud rates. The baud rate of 31,250 bps is the one used by default for the MIDI protocol

For send durations, the main effect of device missed significance [*F*(1, 19) = 3.32, *p* = .08, *η*_G_^2^ = .01]. No other main or interaction effects reached significance (*p*s > .21). These effect sizes were negligible and, as is shown in Fig. 3a, the differences between devices were small (mean difference = 0.0008 ms) and fell within the margin of error of our recording equipment (0.023 ms). Thus, these data were not subjected to planned comparisons. Overall, these results indicate that the baud rate and (by design) the read method do not significantly influence the speed at which MIDI messages are sent. The PCB of the device sending a MIDI message does not appear to have a significant influence on send durations.

For transit durations, all main effects were significant (*p*s < .001, *η*_G_^2^s > .86), all two-way interactions were significant (*p*s < .001, *η*_G_^2^s > .46), but there was no significant three-way interaction (*p* = *.*17). Pairwise comparisons examining the significant interaction between device and method revealed that the “single-byte” method demonstrated significantly smaller transit durations than the “all-bytes” method for both devices (*p*s < .001, mean difference = 0.63 ms). No significant differences emerged between devices for either the “single-byte” method or the “all-bytes” method (*p*s > .99). Planned comparisons investigating the significant interaction between baud rate and method revealed that transit durations decreased significantly as baud rates increased for both the “single-byte” (*p*s < .001) and “all-bytes” (*p*s < .001) methods. Moreover, the “single-byte” method had significantly smaller transit durations than the “all-bytes” method overall (*p*s < .001; see Fig. 3b). Planned comparisons examining the significant interaction between baud rate and device yielded significant differences between devices for each baud rate (*p*s < .001), with the exception of the 15,625 baud rate (*p* = .10, mean difference = 0.002 ms). For both the Arduino Mega and Due, transit durations decreased significantly as baud rates increased (*p*s < .001), with the exception of the 31,250 to 46,875 baud rate (*p*s > .23, mean difference = .21 ms) and 46,875 to 62,500 baud rate (*p*s > .91, mean difference = .11 ms).

For read durations, I found significant main effects of baud rate (*η*_G_^2^ = .999) and method (*η*_G_^2^ = .999), and significant interaction effects between baud rate and method (*η*_G_^2^ = .999), as well as between device and method (*η*_G_^2^ = .02; *F*s > 5.05, *p*s < .04). Pairwise comparisons examining the significant interaction between device and method revealed that the “single-byte” method demonstrated significantly higher read durations than the “all-bytes” method for both devices (*p*s < .001, mean difference = 0.63 ms). Note that this is the reverse trend to that observed for transit durations. There were no significant differences between devices for the “single-byte” and “all-bytes” methods (*p*s = 1.00). Planned comparisons examining the significant interaction between baud rate and method revealed no significant differences between baud rates for the “all-bytes” method (*p*s = 1.00), that read durations significantly decreased as baud rates increased in the "single-bytes" method (*p*s < .001), and that read durations for the “single-byte” method were all significantly longer than those for the “all-bytes” method (see Fig. 3c).

For total durations, all main effects were significant (*p*s < .001, *η*_G_^2^s > .40), all two-way interactions were significant (*p*s < .001, *η*_G_^2^s > .05), and the three-way interaction was not significant (*p* = *.*81). Pairwise comparisons examining the significant interaction between device and method revealed no significant differences (*p*s > .90). Planned comparisons examining the significant interaction between baud rate and method revealed that all conditions were significantly different (*p*s < .001). As is shown in Fig. 3d, the total durations were longer for the “all-bytes” method than for the “single-byte” method and decreased as the baud rate increased. Planned comparisons examining the significant interaction between baud rate and device revealed no significant difference between devices for the 15,625 (*p* = 1.00, mean difference = 0.0008 ms) and 3,1250 (*p* = .25, mean difference = 0.01 ms) baud rates, but significant differences for the 46,875 (*p* = .02, mean difference = 0.018 ms) and 62,500 (*p* = .04, mean difference = 0.018 ms) baud rates. Again, total durations decreased significantly as baud rates increased for both devices.

To test the hypothesis that the total duration of sending and reading MIDI messages is within 1 ms, one-sample, two-tailed *t* tests against the test value 1 (representing 1 ms) were performed on the total duration for each condition. At the lowest baud rate (15,625 bps), total durations were significantly longer than 1 ms (*p*s < .001) for both devices. At the default MIDI baud rate (31,250 bps), the total durations for both devices were significantly less than 1 ms for the “single-byte” method (*p*s < .001), but were significantly longer than 1 ms for the “all-bytes” method (*p*s < .001). The two highest baud rates (46,875 and 62,500 bps) produced total durations that were significantly less than 1 ms for both devices and methods (*p*s < .001). Thus, MIDI messages could only be sent and read within 1 ms for baud rates of 46,875 bps and higher, or for the default MIDI baud rate (31,250 bps) when the “single-byte” method was used.

### Discussion

The results here demonstrated that both the transit and read durations are affected by the read method and baud rate, in turn affecting the total duration. Contrary to my hypothesis, the send duration of MIDI messages was relatively unaffected by differences in device and baud rate. However, the hypothesis that read and transit durations are influenced by read method and baud rate was supported. When MIDI messages were read as they were received (instead of waiting for the entire three-byte message), the transit and total durations improved significantly. Conversely, read durations were larger for the “single-byte” method than for the “all-bytes” method because the read duration of the former included the time waiting for the second and third bytes to be received (i.e., a portion of the transit duration). The hypothesis that the “single-byte” method would produce significantly shorter transit and total durations than the “all-bytes” method was confirmed. This result suggests that the “single-byte” method produces lower latencies for receiving MIDI messages and could be implemented by devices that read MIDI for superior performance (if this method is not already implemented). However, these performance gains were modest relative to increasing the baud rate. The hypothesis that total durations would decrease as the baud rate increases (i.e., the higher the bps) was supported, with total latencies being almost halved when the baud rate was doubled from the MIDI standard (i.e., from 31,250 to 62,500 bps). These results indicate that one of the bottlenecks for high-speed MIDI communication is the baud rate. In fact, the total duration for MIDI messages did not consistently fall below the suggested 1-ms duration (Finney, 2001; Kierley, 1991) until the baud rate was increased to either 46,875 or 62,500 bps; the native MIDI baud rate of 31,250 only fell below 1 ms for the “single-byte” method (although the range of 0.79 to 1.07 ms included 1 ms; see Appendix 2, Table 7). These results indicate that the transmission duration of MIDI messages could be substantially improved by implementing adjustable baud rates for MIDI devices. The average user would probably not be able to make these changes to MIDI devices themselves, so it is up to the developers of such devices to make this a built-in option. Specifically, developers could include the option to select higher baud rates within the device interface and ensure that these baud rates are supported for future MIDI devices.

## Experiment 2: MIDI–PCI and MIDI–USB interfaces

Experiment 2 examined the transit duration for MIDI messages from the time they are sent to the time they are first read when being directed through a MIDI–PCI or MIDI–USB interface (see Fig. 1b). The latency and variability of six MIDI interfaces were tested, three of which were MIDI–PCI interfaces and three of which were MIDI–USB interfaces to test whether MIDI–PCI interfaces produce shorter and less variable latencies than MIDI–USB interfaces. Finally, I tested whether MIDI–USB devices poll—that is, is the USB port periodically sampled to see if new information has arrived at intervals greater than 1 ms.

### Method

#### Materials

The “send” and “read” Arduino triggers (audio data) were recorded using the same setup as Experiment 1. An Intel Core i7-2670QM, 2.2 GHz, running Linux Ubuntu v3.2.0-23 was used to perform the FTAP loop test (Finney, 2001).

#### Procedure

Latencies were measured using the SMIDIBT route test, which sends 4,002 MIDI “note on” and “note off” messages and compares the sent time with the received time. Experiment 1 indicated that transit durations were shorter for the “single-byte” read method. Accordingly, that method was used here to record the transit and total durations. As in Experiment 1, sent and received times are demarcated, respectively, by a change from high to low for the “send Arduino” and by a change from low to high for the “read Arduino.” The difference between these times indicates the transit duration for the MIDI message. When routed through a MIDI–PCI or MIDI–USB, the transit duration indexes the latencies incurred when a PC receives and sends a MIDI message. The onset and offset times used to measure transit durations were recorded at a sampling rate of 44100 Hz, allowing a temporal resolution of 0.023 ms. The SMIDIBT route test was conducted 20 times for each device (6) by interval (3) combination. The three interval rates represented different information loads: 1, 2, and 3 ms between sends. Moreover, a baseline was calculated on the basis of the latencies produced when the MIDI messages were not routed through an interface but were, instead, directly sent from the “send Arduino” to the “read Arduino” (as in Exp. 1). To measure polling, the FTAP loop test was used, which sends 4,002 MIDI messages through a MIDI device that connects its own output to its input and records the latency between the sent and received times (rounded to the nearest millisecond). I then assessed whether the distribution of latencies was bimodal or unimodal. A bimodal distribution would be indicative of polling, whereas a unimodal distribution would indicate either that the data transfers were not subject to polling or they polled at a rate that was distributed around one central latency value. The FTAP loop test was conducted 20 times per device.

#### Design and hypotheses

The dependent variables were the raw latencies and the standard deviation (*SD*) of latencies for each loop test. To better reflect the typical standard deviations found in sensorimotor synchronization experiments, the *SD* of each group of 40 consecutive events was calculated. The FTAP loop test further included Hartigan’s distribution statistic as a dependent variable for measuring multimodality for each trial (Hartigan & Hartigan, 1985). The independent variables were the interval (1, 2, 3 ms; SMIDIBT route test only^1^) the device model (six levels: LogiLink MIDI–USB, M-Audio UNO, Roland UM-ONE, Labway Soundboard D66, Sound Blaster Live, TerraTec TT Solo 1-NL). The former three models were MIDI–USB interfaces, and the latter three were PCI devices. Following the suggestions of Finney (2001), I hypothesized that the three MIDI–PCI cards would produce lower and less variable latencies than the MIDI–USB interfaces. I also hypothesized that the latencies produced by the three MIDI–PCI cards would produce a unimodal distribution, whereas the MIDI–USB devices would produce a multimodal distribution if they poll (or a unimodal distribution if they do not poll). I further hypothesized that the MIDI messages would be read and sent within 1 ms.

#### Statistical analyses

Since there were unequal variances between devices, the data were analyzed using linear mixed-effects models (LMEM) with the fixed factors device and interval and a random effect of repetition (20 levels), and unequal variances were permitted across the levels of the device factor. The model was fit using the lme function from the nlme library (Pinheiro, Bates, DebRoy, Sarkar, & R Core Team, 2015) for the R package of statistical computing (R Core Team, 2013), and unequal variances were implemented using the varIdent model formula term. Pair-wise contrasts were computed using generalized linear hypothesis testing for Tukey contrasts, using the glht function in the multcomp library (Hothorn et al., 2008). The LMEM was used to analyze the dependent variables latency and variability. Bayes factors were calculated to test the probability that the null hypothesis could be accepted (less than 1) or rejected (greater than 1; Rouder, Speckman, Sun, Morey, & Iverson, 2009). The Bayes factor is an odds ratio, but I adopt the nomenclature used by Jeffreys (1961) that values around 1 suggest “no evidence,” values between 1 and 3 suggest “anecdotal evidence,” those between 3 and 10 suggest “substantial evidence,” those between 10 and 30 suggest “strong evidence,” those between 30 and 100 suggest “strong evidence,” and those over 100 suggest “decisive evidence.” Moreover, I represent evidence for the alternative hypothesis as BF_HA_, and evidence for the null hypothesis as BF_H0_ (i.e., 1/BF). The Bayes factor was computed using the ttestBF function in the BayesFactor library (Morey, Rouder, & Jamil, 2009).

Multimodality of latency distributions was assessed using Hartigan’s dip test for unimodality (Hartigan & Hartigan, 1985), where values lower than .05 indicate unimodality and values greater than 0.05 indicate multimodality. The FTAP loop test only measures latencies to the nearest millisecond and, with the resulting range of 0 to 3 ms, this provided up to four bins per condition, thus failing to meet the recommended number of bins 1 + log_2_(*N*), or about 13 bins for the 4,002 data points produced in each loop test (see Sturges, 1926). Since Hartigan’s dip test could not be performed on the rounded raw data, a resampling method was employed whereby a uniform distribution of random numbers between – .49 and .49 were added to the raw data and any values less than zero were made positive. This method was chosen because it reflects both the possible latency values prior to rounding and the mean delay between output scheduling calls reported by FTAP during the FTAP loop test (0.49 ms). Moreover, a send/receive delay of zero is both theoretically and practically impossible given that MIDI messages require approximately 1 ms to be transmitted (Kierley, 1991), as demonstrated in Experiment 1. A uniform distribution was used because a normal distribution would have favored a multimodal distribution. Thus, the use of a uniform distribution is more conservative given our hypothesis of multimodality for the MIDI–USB devices. Five different random distributions were applied to the latencies of the six devices for each of the 20 repetitions and the Hartigan’s dip test statistic was calculated for each repetition by random distribution combination using the dip.test function in the diptest library (Maechler, 2015). The resulting distribution statistics were compared to the critical value of .05 using one-sample two-tailed *t* tests.

### Results

#### SMIDIBT route test

**Fig. 4:**
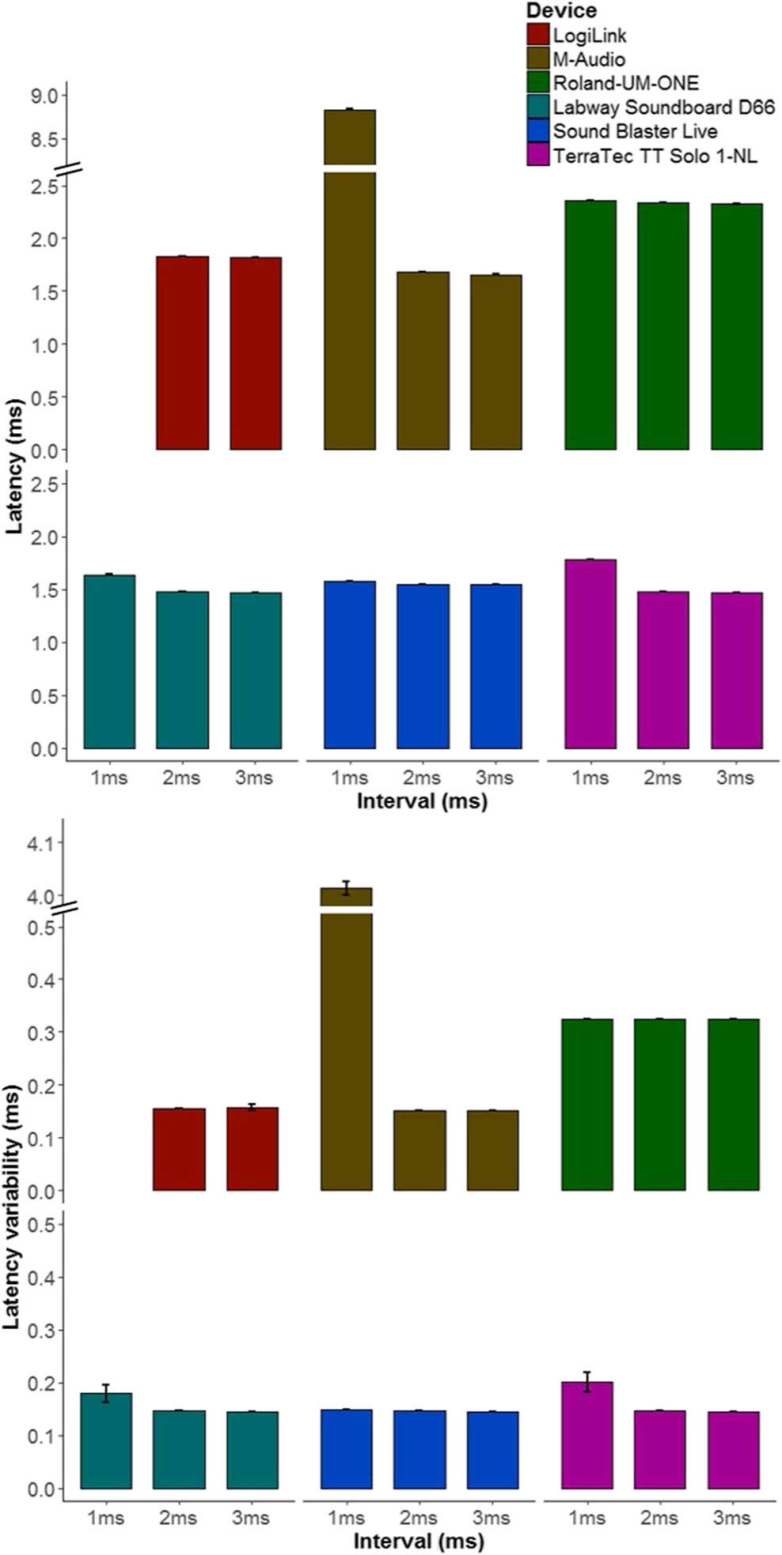
Mean latencies (top panel) and variability (bottom panel) in the SMIDIBT route test (Exp. 2). Error bars represent standard errors of the means

In the 1-ms condition, the LogiLink device was unable to complete any trial without an error, and the M-Audio demonstrated a constant drift, with transit durations monotonically increasing in each trial. For these reasons, two LMEM analyses were conducted; one with the 1-ms interval condition removed and another with the LogiLink device removed. For latencies, both the interactions excluding the 1-ms interval [*F*(5, 480240) = 941.97, *p* < .001, *η*_G_^2^ = .99] and excluding the LogiLink device [*F*(5, 480240) = 941.97, *p* < .001, *η*_G_^2^ = .99] were significant (*p*s < .001), so I proceeded with pair-wise comparisons. All conditions were significantly different from each other, with the exception of the following: The TerracTec and Labway were not significantly different for the 2-ms and 3-ms intervals, and there were no significant differences between the 2-ms and 3-ms intervals for the LogiLink and Sound Blaster. As is shown in Fig. 4, the 1-ms interval produced higher latencies than the 2-ms and 3-ms intervals for all devices (*p*s < .001). The 2-ms interval only produced significantly larger latencies than the 3-ms interval for the M-Audio (*p* < .001) and Roland UM-ONE (*p* = .03). For the 1-ms interval (i.e., high information load), the Sound Blaster had the lowest latency, followed by the Labway, TerraTec, Roland UM-ONE, and M-Audio (*p*s < .001). For the both the 2-ms and 3-ms intervals, the Labway and TerraTec had the lowest latencies (*p*s = 1), followed by the Sound Blaster, M-Audio, LogiLink, and Roland UM-ONE (*p*s < .001). These results support the hypothesis that MIDI–PCI interfaces have significantly lower latencies than MIDI–USB interfaces. Finally, to test whether 1-ms latencies could be achieved, the mean baseline value for each interval was subtracted from each latency within that interval, and one-sample, two-tailed *t* tests were performed against the test value 1. For all devices and intervals, the latencies were significantly greater than 1 ms (*p*s < .001, *df*s = 19). These results fail to support the hypothesis that 1-ms performance is achievable for either MIDI–PCI or MIDI–USB interfaces. For descriptive statistics related to the route test, see Appendix 3.

For variability, two LMEM analyses were conducted, separately excluding the 1-ms interval and then excluding the LogiLink (as was performed for latencies). The LMEM with the 1-ms interval excluded only showed a significant main effect of device [*F*(5, 85) = 3587.47, *p* < .001, *η*_G_^2^ = .988], whereas the LMEM with the LogiLink excluded demonstrated significant main effects of device and interval (*p*s < .001, *η*_G_^2^s > .99), as well as a significant two-way interaction [*F*(8, 280) = 19,823, *p* < .001, *η*_G_^2^ = .998]. On the basis of these results, I proceeded with planned comparisons only between the 1-ms interval and the 2-ms and 3-ms intervals for each device and between devices. Only the Labway, TerraTec, and M-Audio had significantly greater variability for the 1-ms interval compared to the 3-ms interval (*p*s < .04), and only the TerraTec and M-Audio had significantly greater variability for the 1-ms interval compared to the 2-ms interval (*p*s < .001; all other *p*s > .99). As is shown in Fig. 4 (bottom panel), for the 1-ms interval, the PCI interfaces (Sound Blaster, Labway, and TerraTec) all had the least variability (*p*s > .10), followed by the Roland UM-ONE (*p*s < .001), whereas the M-Audio was the most variable (*p*s < .001). For the 2-ms and 3-ms intervals, the Roland UM-ONE was significantly more variable than all other devices (*p* < .001), and no other devices demonstrated significant differences (*p*s > .99). These results partially support the hypothesis that PCI–USB interfaces are less variable than MIDI–USB interfaces; PCI–USB interfaces are only less variable than MIDI–USB interfaces when under high information load—that is, the 1-ms interval condition.

#### FTAP loop test

For FTAP loop test latencies, there was a significant main effect of device [*F*(5, 480240) = 941.97, *p* < .001, *η*_G_^2^ = .99]. Pair-wise comparisons yielded significant differences between devices (*p*s < .001), with the exceptions of between the M-Audio UNO and the three MIDI–PCI devices (*p*s > .83) and between the three PCI devices (*p*s > .86). As is shown in Table 2, the three PCI devices and the M-Audio UNO were significantly faster, followed by the Roland UM-ONE, and the LogiLink MIDI–USB was the slowest. Bayes factor *t* tests between the M-Audio UNO and PCI devices suggested substantial evidence for the null hypothesis for the Sound Blaster Live card (BF_H0_ = 8.12), and extreme evidence for the null hypothesis for the TerraTec TT and Labway Soundboard (BF_H0_s > 245.5). Bayes factor *t* tests between the three PCI devices revealed strong evidence for the null hypothesis between the Labway and Sound Blaster Live (BF_H0_ = 17.54) and between the Sound Blaster Live and TerraTec (BF_H0_ = 20.61), and extreme evidence for the null hypothesis between the Labway and TerraTec (BF_H0_ = 249.83).

**Table 2:** Descriptive statistics for FTAP loop latency, variability, and Hartigan’s *D* statistic

Dependent Variable	Interface	Device	Mean	*SD*	Min	Max
Latency (ms)	USB	LogiLink MIDI–USB	1.223	1.271	0	4
		M-Audio UNO	0.964	0.989	0	3
		Roland UM-ONE	1.001	1.023	0	4
	PCI	Labway Soundboard D66	0.963	0.799	0	2
		Sound Blaster Live	0.958	0.795	0	2
		TerraTec TT Solo 1-NL	0.964	0.799	0	2
Variability (ms)	USB	LogiLink MIDI–USB	1.271	0.001	1.269	1.273
		M-Audio UNO	0.989	0.001	0.987	0.991
		Roland UM-ONE	1.023	0.022	1.001	1.049
	PCI	Labway Soundboard D66	0.799	0.002	0.796	0.803
		Sound Blaster Live	0.795	0.010	0.779	0.810
		TerraTec TT Solo 1-NL	0.799	0.003	0.793	0.805
Hartigan’s *D* stat.	USB	LogiLink MIDI–USB	0.083	0.000	0.082	0.083
		M-Audio UNO	0.100	0.001	0.099	0.103
		Roland UM-ONE	0.107	0.018	0.086	0.126
	PCI	Labway Soundboard D66	0.005	0.001	0.003	0.008
		Sound Blaster Live	0.005	0.001	0.003	0.009
		TerraTec TT Solo 1-NL	0.005	0.001	0.003	0.010

For variability, I observed a significant main effect of device [*F*(5, 120) = 7,865.6, *p* < .001, *η*_G_^2^ = .99]. Pair-wise comparisons yielded significant differences between devices (*p*s < .001), with the exception of the three PCI devices (*p*s > .71). As is shown in Table 2, the Sound Blaster Live card was the least variable, then the Labway and TerraTec, then the M-Audio UNO and the Roland UM-ONE, and the LogiLink was the most variable. Bayes factor *t* tests between the three PCI devices revealed anecdotal evidence for the null hypothesis between the devices (BF_H0_s < 2.65).

**Fig. 5:**
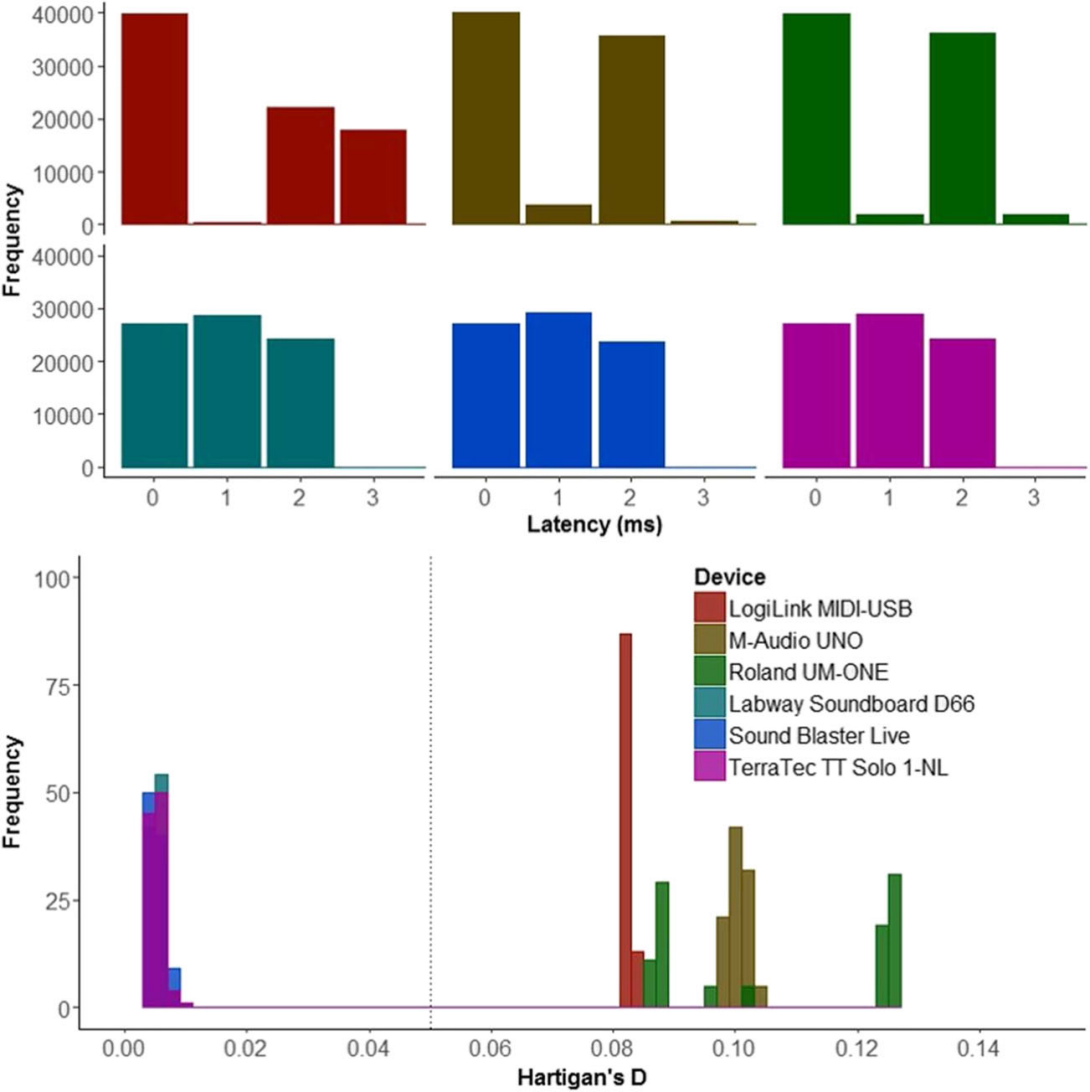
Histograms of latencies (top panel) and Hartigan’s distribution statistics (bottom panel) in the FTAP loop test. The dashed vertical line in the bottom panel represents the critical value for unimodality (less than .05) versus multimodality (greater than .05)

Regarding unimodality, all three PCI cards demonstrated Hartigan’s distribution statistics that were significantly less than the critical value for multimodality (.05) [*t*s < – 397.4, *p*s < .001], indicating a unimodal distribution (see Fig. 5). Conversely, all MIDI–USB devices showed Hartigan’s distribution statistics that were significantly greater than the critical value for multimodality (.05) [*t*s > 31.9, *p*s < .001], indicating multimodal distributions. Note that none of the resampling methods produced values approaching .05 for the PCI devices, and that all of the resamples were greater than .05 for the MIDI–USB devices (see Fig. 5).

### Discussion

The results of Experiment 2 indicated that the three MIDI–PCI cards have lower latencies than the MIDI–USB interfaces (except for the M-Audio UNO in the FTAP loop test). Moreover, the MIDI–PCI cards were less variable than the MIDI–USB interfaces but only when placed under high information load (i.e., the 1-ms interval of the SMIDIBT route test and in the FTAP loop test). I also found support for the hypothesis that the MIDI–USB interfaces poll as suggested by the multimodal distributions given by the latencies these devices and further corroborated by the lack of polling for the PCI cards. These results support the statements of Finney (2001) that suggested that MIDI–PCI cards produce smaller latencies than alternative options. Polling of the MIDI–USB devices could be a driving factor of the poorer performance of these devices as compared to MIDI–PCI devices. In the SMIDIBT route test, latencies were significantly above the 1-ms resolution reported by Finney (2001), regardless of the interval or interface type. In the FTAP loop test, the average sub–millisecond latencies reported by Finney were replicated here for the MIDI–PCI cards, and also for the M-Audio UNO. However, the latency values for the MIDI–PCI cards ranged from 0 to 2 ms, the lower limit being theoretically impossible, given that serial MIDI messages are not instantaneous (Kierley, 1991), and the upper limit remaining above the within-1-ms resolution that is sometimes assumed for MIDI interfaces. Thus, even using nonpolling MIDI–PCI devices does not consistently produce sub-millisecond latencies when recording responses, and MIDI–PCI interfaces are only significantly less variable than MIDI–USB interfaces under high information load.

The present study only involved FTAP software because it had been found to produce lower and less variable latencies than Max/MSP in a previous experiment (Schultz & van Vugt, 2016). Other programs are available (e.g., Max/MSP, Python) with which one could make custom scripts for parsing MIDI messages. Since there are no standard scripts for parsing MIDI messages using these programs, there could be variations in the latencies produced by the different scripts. Future studies could use the SMIDIBT to examine differences between custom scripts and various software packages used to parse MIDI messages. Moreover, the effect of different computer operating systems on MIDI latencies could be benchmarked using cross-platform software, such as Max/MSP (available for Windows or Macintosh OS) or Python (available for Windows, Macintosh, or Linux OS).

## Experiment 3: MIDI percussion pads

In Experiment 3 I investigated the audio and MIDI latencies that occur when a MIDI percussion pad is struck by a finger (see Fig. 1c). To establish the veridical onset times of responses and auditory feedback in each setup, I recorded finger onset times using a force-sensitive resistor (FSR) attached to the drum pad (as in Schultz & van Vugt, 2016). Response onsets (i.e., FSR voltage changes), percussion pad audio onsets, and MIDI trigger onsets from the “read Arduino” were recorded simultaneously in a synchronized manner using an external audio sound card. The sound card recorded voltage readings from the FSR, on which participants tapped. Auditory feedback and MIDI messages from the various MIDI percussion pad devices were also captured by the sound card. Because Schultz and van Vugt (2016) demonstrated that harder taps resulted in significantly lower latencies and less variability of the auditory feedback asynchrony for the MIDI percussion pad, all taps were performed with a hard force. Note that the aim of Experiment 3 was to show that the SMIDIBT can be used to benchmark the audio and MIDI latencies of percussion pads; missed and duplicate responses for these devices and the results of different tapping forces were beyond the scope of this study.

### Design and hypotheses

The dependent variables were the asynchronies between (1) the response onset and the audio onset, (2) the response onset and the “read Arduino” onset (i.e., the MIDI message reception time, excluding the read time), and (3) the audio onset and the “read Arduino” onset. The independent variable was device, consisting of the three MIDI percussion pads—namely, the Alesis Percussion Pad (PercPad), the Roland Sampling Percussion Drumpad 6 (SPD6), and the Roland Handsonic Hand Percussion Drumpad 20 (HPD20). These devices were chosen because the former represents a less expensive percussion pad (US $199), and the latter two devices represent the percussion pads used in previous sensorimotor synchronization experiments (see Table 1) but are considerably more expensive; the SPD6 has been discontinued, but the SPD-SX has a manufacturer’s suggested retail price of US $1,000, and the HPD20 has a recommended retail price of US $1,049. On the basis of the previous performance of percussion pads (Mills et al., 2015; Schultz & van Vugt, 2016), I hypothesized that both the audio and MIDI latencies would be significantly larger than 1 ms and that the audio and MIDI onsets would occur asynchronously. Regarding percussion pad devices, it was hypothesized that some devices might be more reliable than others (i.e., lower latencies, less variability), due to differences in hardware and software configurations that are unspecified by the manufacturer.

### Method

#### Materials

Experiment 3 used the same equipment as the previous two experiments, with the exception that a Focusrite Scarlett 18i8 mixer was used to synchronize and record the responses, audio, and “read Arduino” signals.^2^ Response onsets were recorded using an Interlink square FSR (3.81cm) powered by an Arduino UNO R3 with the direct voltages sent to an audio jack (see Schultz & van Vugt, 2016; van Vugt & Schultz, 2015). Although Experiment 1 had shown no significant differences between the Arduino Mega and Due, the “read Arduino” of the SMIDIBT was an Arduino Due (see Fig. 2), which has a faster clock speed (84 MHz). The FSR was placed on the center of the bottom right (i.e., one of the largest drum surfaces) of each percussion pad. The MIDI onsets of the percussion pads were delivered from the MIDI OUT port of the percussion pad to the MIDI IN of the “read Arduino,” and the percussion pad audio was sent from the mono audio out port. The FSR voltages, “read Arduino” triggers, and auditory signals were measured by the Focusrite mixer at a sampling rate of 44100 Hz. The voltage changes produced by applying pressure to the FSR were simultaneously recorded by the mixer in order to synchronize responses with the auditory feedback.

#### Auditory stimuli

Each percussion pad comes with a range of audio patches. For this reason, I tested several patches for each percussion pad, excluding those that were sound effects, symbols, or instruments with no clear onset or offset (e.g., rain sticks, wind chimes, vibraslap). Moreover, because the HPD20 contains about 850 patches, only a selection of dissimilar patches were tested, and duplicate sounds (i.e., variations of the same instrument) and most “rim hits” were excluded. For tonal percussion instruments, only those with the highest pitch were chosen. As I stated earlier, the aim of Experiment 3 was not to exhaustively test all MIDI instrument patches, but was instead to measure MIDI latencies and introduce the SMIDIBT so that researchers can test the audio and MIDI latencies in their own devices. To optimize response detection by the percussion pad and make audio onset detection easier, the devices were configured such that all effects and reverb were turned off, sensitivity levels were set to maximum, and threshold levels were set to minimum. The HPD20 and SPD6 also contained options for controlling the audio output that were implemented; the velocity curve was set to “Loud2” and “Loud” (high volume from the outset for every response) for the HPD20 and SPD6, respectively. For the HPD20, the trigger mode was set to “Shot” (short duration), and muffling (i.e., reducing the sound envelope tail) was set to maximum (100).

### Results

#### Onset extraction

Voltage changes from the FSR and audio onsets were extracted using a custom-made MATLAB script (available from https://band-lab.com/smidi_toolbox). An FSR onset was measured as the time for which the normalized amplitude (range – 1 to 1) exceeded .02 and another onset was not detected until 60 ms after the amplitude had descended below – .02 (indicating FSR depression). For each FSR onset, the corresponding audio onset was the first sample at which the absolute normalized amplitude exceeded 0.1. To further aid in audio onset extraction, the data were smoothed such that each sample of the audio signal contained the maximum value of the preceding 44 samples (approximately 1 ms), except for the first 44 samples of each recording, in which no onsets occurred.^3^ MIDI onset times were measured using the SMIDIBT “read Arduino” method, as in Experiment 1. Any responses that produced no auditory signal or MIDI note onset within 20 ms after the FSR onset were discarded. Data collection continued until at least 200 audio onsets had been extracted for each audio patch tested.

#### Statistical analysis

Due to the large number of audio patches tested and the fact that these patches likely varied between instruments, I do not present all individual pairwise comparisons between these instruments. Instead, the means, standard deviations, ranges, standard errors of the means, and 95% confidence intervals are provided in the files associated with Supplementary Materials 2, 3 and 4, and I only examine the audio that provided the lowest and highest latencies for each device. To deal with the problem of unequal data points and variances, a LMEM was fit to the data, with the fixed factors latency group (two levels: lowest latency, highest latency) and device (three levels: PercPad, HPD20, SPD6), and the random factor response (approximately 200 levels). I allowed unequal variances across the levels of device and latency group, which was decided on the basis of visual observation that the residuals were highly nonhomogeneous for the various devices and instruments. The model was fit using the lme function of the nlme library (Pinheiro et al., 2015) for the R package of statistical computing (R Core Team, 2013), and unequal variances were implemented using the varIdent model formula term. The LMEM was used to analyze all dependent variables.

#### FSR-to-audio latencies

**Fig. 6:**
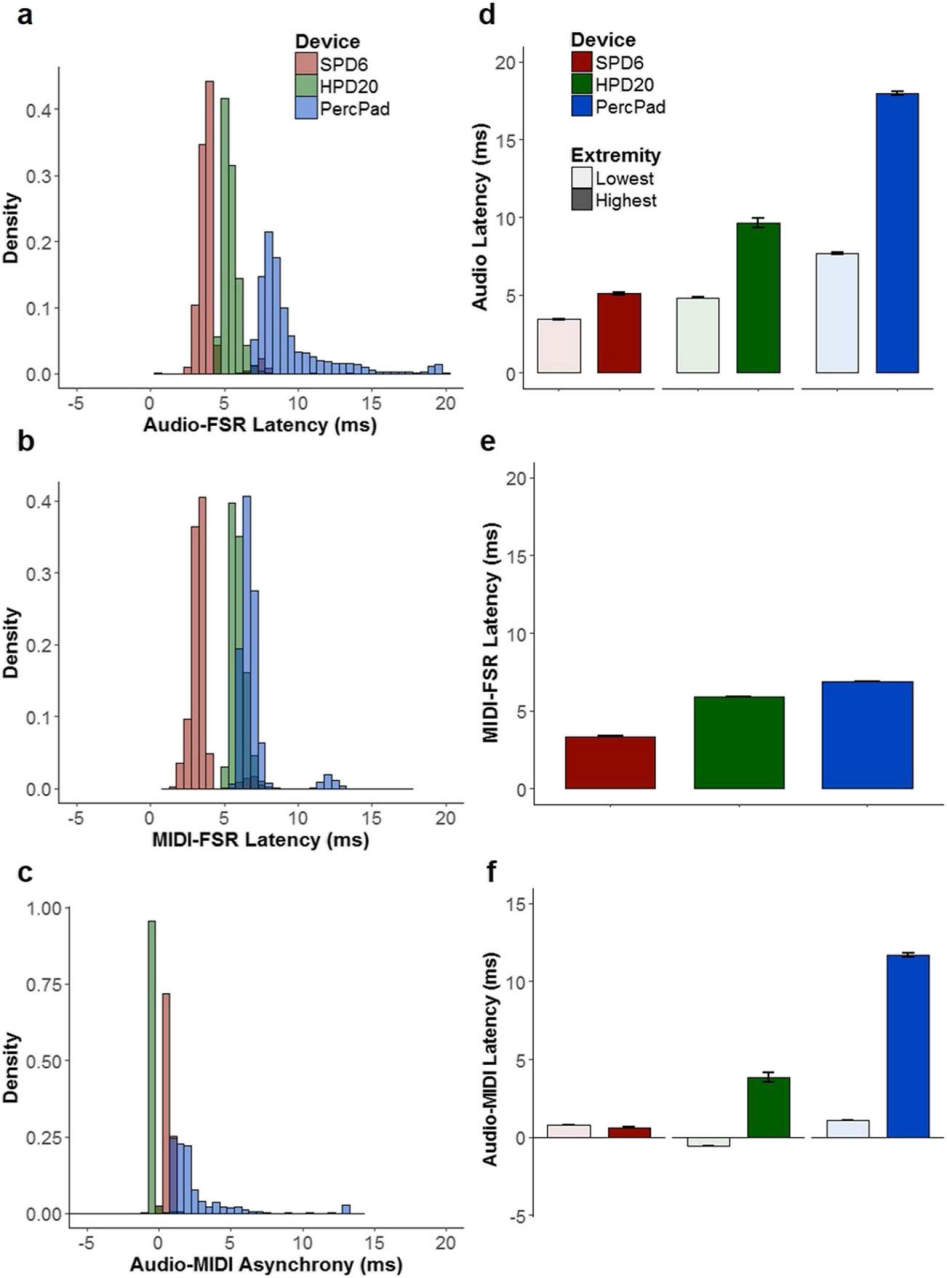
Left column: Histograms of the latency between the FSR and audio (a; audio onset minus the FSR onset), the FSR and MIDI received trigger (b; MIDI trigger onset minus the FSR onset), and the audio and MIDI trigger (c; audio onset minus the MIDI trigger onset). Right column: Latencies for instruments with the lowest and highest latencies for all drum pad devices. (d) Latencies between the FSR and audio (audio onset minus the FSR onset). (e) Latencies between the FSR and MIDI trigger (MIDI trigger onset minus the FSR onset). (f) Latencies between the audio and MIDI trigger (audio onset minus the MIDI trigger onset). Error bars represent standard errors of the mean

Figure 6a shows histograms of the FSR-to-audio latencies for all instruments and devices. For the PercPad, the instruments with the lowest and highest latencies were the “E Snare Hex” and “Tabla High,” respectively. For the HPD20, the instruments with the lowest and highest latencies were “Clap 1” and “Bendir Harm,” respectively. For the SPD620, the instruments with the lowest and highest latencies were the “808 Kick 1” and “808 High Tom 1,” respectively. I found significant main effects of device [*F*(2, 1510) = 5,308.32, *p* < .001, *η*_G_^2^ = .81] and latency group [*F*(1, 1510) = 4,691.26, *p* < .001, *η*_G_^2^ = .74], as well as a significant interaction between device and latency group [*F*(2, 1510) = 1,196.59, *p* < .001, *η*_G_^2^ = .50]. Pair-wise comparisons revealed significant differences between all devices and latency groups (*p*s < .001), with the exception of the lowest-latency instrument of the HPD20 and the highest-latency instrument of the SPD6 (*p* = .15). As is shown in Fig. 6d, the SPD6 demonstrated the lowest latencies overall, followed by the HPD20, then the PercPad. Similarly, the difference between the lowest- and highest-latency instruments was smallest for the SPD6, followed by the HPD20, and then the PercPad. One-sample, two-tailed *t* tests against a test value of 1 (representing 1 ms) revealed that all latencies were significantly greater than 1 ms (*p*s < .001, *df*s = 200). Thus, the hypothesis that the latency of auditory feedback after a response would be greater than 1 ms was supported.

#### FSR-to-MIDI latencies

Figure 6b shows histograms of the FSR-to-MIDI latencies for all devices and instruments. There was a significant main effect of device [*F*(2, 55854) = 24,728.90, *p* < .001, *η*_G_^2^ = .97]. Pair-wise comparisons revealed significant differences between all devices (*p*s < .001). As is shown in Fig. 6e, FSR-to-MIDI latencies were shortest for the SPD6, followed by the HPD20, and the PercPad produced the highest latencies. The results indicate that devices differ in the speed at which MIDI messages are sent following a response. To test the hypothesis that MIDI messages would be sent within 1 ms of a response, one-sample, two-tailed *t* tests were performed against the test value of 1. All devices demonstrated FSR-to-MIDI latencies that were significantly greater than 1 ms (*p*s < .001). Thus, the hypothesis that MIDI messages would be sent more than 1 ms after a response was supported.

#### MIDI-to-audio latencies

Figure 6c shows histograms of the MIDI-to-audio latencies for all devices and instruments. Regarding the instruments with the lowest and highest latencies for each device, significant main effects emerged for device [*F*(2, 1479) = 27,943.58, *p* < .001, *η*_G_^2^ = .60] and latency group [*F*(1, 1479) = 127.81, *p* < .001, *η*_G_^2^ = .62], as well as a significant interaction between device and latency group [*F*(2, 1479) = 4,057.97, *p* < .001, *η*_G_^2^ = .55]. Pair-wise comparisons revealed significant differences between all devices and latency groups (*p*s < .001). As is shown in Fig. 6f, all devices and instruments showed positive latencies, with the exception of the lowest-latency instrument for the HPD20. This indicates that, for the SPD6 and PercPad, the MIDI message was sent (and received) prior to the production of audio. As is shown in Fig. 6c, the HPD20 tended to produce the audio before the MIDI message was sent (and received), and the highest-latency instrument for this device is not representative of the general performance for the instrument of the HPD20. Interestingly, the SPD6 demonstrated an opposite trend from all other devices, so that the audio for the instrument with the lowest latency was more synchronous with the MIDI signal than that for the instrument with the highest audio latency. This result indicates that the MIDI signal latency might also be affected by the MIDI instrument playing on the actual device.^4^ Overall, these results indicate that the audio onset is asynchronous with the MIDI onset and that devices differ in how they prioritize the audio and MIDI signals.

### Discussion

In Experiment 3 I examined three MIDI percussion pads. In line with my hypothesis (based on previous experiments that had tested MIDI percussion pads), none of the MIDI percussion pads produced MIDI signals or audio consistently within 1 ms of a response. The SPD6 produced the lowest MIDI latencies but still had a mean latency of 3.35 ms. The PercPad produced the highest latencies, and since it was the least expensive of the devices by several hundred US dollars, this suggests that the more expensive commercially available MIDI percussion pads tested here provide superior performance. Overall, these results indicate that millisecond resolution may not be achievable when collecting responses with MIDI percussion pads. The SMIDIBT could be used to determine whether such a device exists and can be used to benchmark any MIDI percussion pad or other MIDI device that provides a MIDI “out” port. Future research could investigate the reliability of MIDI percussion pads for accurately recording responses without missing responses or recording duplicate responses, and also examine the effects of different tapping forces (see Schultz & van Vugt, 2016).

## Experiment 4: MIDI sound modules

Another possible source of delay for presenting response-triggered auditory feedback using MIDI is the sound module that receives the MIDI message and generates the feedback (see Fig. 1d). In Experiment 4 I investigated the audio latencies of several MIDI sound modules and a subset of available instruments—specifically, the instruments that produced the lowest and highest latencies, and two instruments that shared a common label between all devices.

### Design and hypotheses

The dependent variable was the asynchrony between the “send Arduino” MIDI trigger offset and the audio onset. The independent variables were the device and latency group. Statistical analyses were not performed on all of the data due to the large number of instruments within each device, and the fact that these sounds might not be equivalent between devices. Instead, these data are reported in full in the file associated with Supplementary Material 5; as well as the lowest and highest latencies were analyzed, and the only two instruments that were common for all sound modules and assumed to be the original GM standard. I hypothesized that devices would differ in the auditory feedback latencies for both the lowest- and highest-latency instruments. I further hypothesized, on the basis of the assumption that the two MIDI instruments should be of the GM standard, that devices would produce similar latencies (i.e., the null hypothesis) for each of the GM instruments tested (harpsichord and vibraphone).

### Method

#### Materials

The materials were identical to those used in Experiment 3. The MIDI sound modules that were tested were two independent sound modules, the Roland Mobile Studio Canvas SD-50 (R-SD50) and the MIDI-Tech Pianobox (MT-PB). These two devices were chosen because the former represents what previous sensorimotor synchronization experiments have used (see Table 1), and the latter represents a less expensive option (approximately US $90 for the same model, but renamed the “MIDIPlus S-Engine”). The R-SD50 uses the Roland GS MIDI, and the MT-PB uses the standard GM. Since some researchers have used the sound module integrated into piano keyboards (see Table 1), I also examined two keyboards—namely, the Korg Micro X synthesizer (K-MX) and a Kawai CL25 Digital Piano (K-CL25), both of which contain GM sound banks that were examined here.

#### Procedure

For each instrument of each device, 201 repetitions were performed and the first onset was excluded (some devices produced the previous instrument on the first onset after a device change). Each sound had a 20-ms duration, but due to differences in sound modules, some sounds continued beyond the designated offset, despite the fact that reverb effects were turned off. As such, a delay of 3,607 ms was used between onsets, to ensure that the previous sound had ended before the next onset commenced. Any instrument that continued to play at the start of the next instrument was removed from the analysis. The same audio onset extraction method was used as in Experiment 3. It should be noted that the MT-PB produced a consistent buzz, and for all devices, some instruments were too quiet to accurately allow for detecting onsets above the baseline noise. For this reason, instruments for which the onset detection algorithm failed (on the basis of visual observations of the plotted data) were excluded from further analysis. The “send Arduino” MIDI trigger offsets were extracted in the same way as in Experiments 1 and 2.

### Results

#### Statistical analysis

As in Experiment 3, I do not present analyses on all of the instruments, and instead the means, standard deviations, ranges, standard errors of the means, and 95% confidence intervals are provided in a .csv file, referenced in Supplementary Material 5. Two analyses were performed: In the first, I only examined the audio that provided the lowest and highest latencies for each device. In the second, I only analyzed the two instruments that shared an identical name between all devices—namely, the harpsichord and vibraphone (but see the Supplementary Material 5 file for descriptive statistics for other instruments with similar names). For the first analysis, an LMEM was fit to the data, with the fixed factors latency group (two levels: lowest latency, highest latency) and device (four levels: K-CL25, K-MX, MT-PB, R-SD50), and the random factor trigger (200 levels). The same LMEM was fit in the second analysis, but replacing the fixed factor latency group with instrument (two levels: harpsichord, vibraphone). I allowed unequal variances across the levels of device on the basis of the visual observation that the residuals were nonhomogeneous for the four devices.

#### MIDI-to-audio latencies

**Fig. 7:**
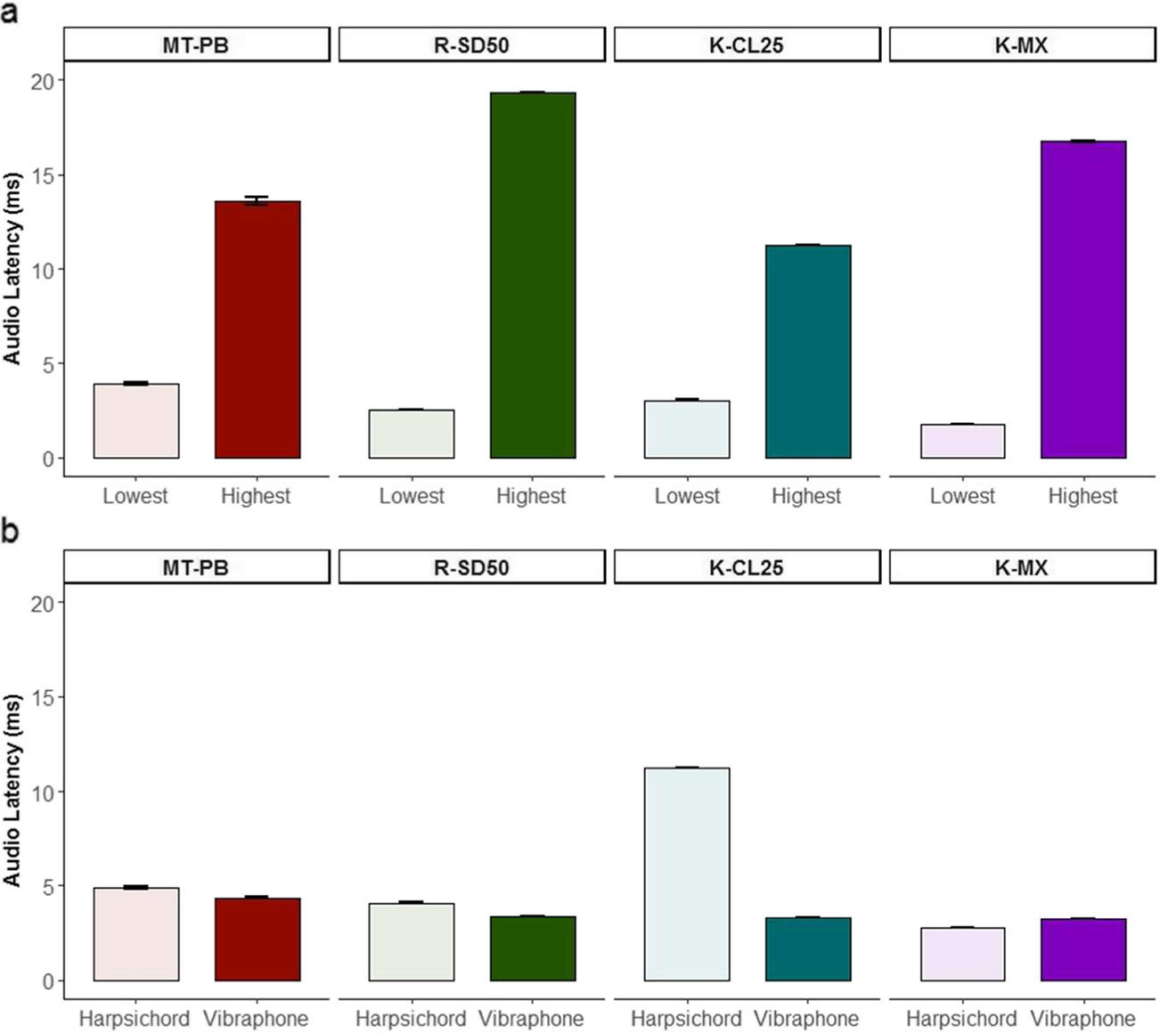
Latencies between the send MIDI offset and audio onsets for the MIDI sound modules. The left panel (a) shows the lowest and highest latencies for each sound module. The right panel (b) shows the latencies for the harpsichord and vibraphone for each sound module. Error bars represent standard errors of the mean

The instruments identified as having the lowest and highest mean latency for each device are shown in Table 3. Descriptive and inferential statistics for all devices and instruments are provided in the Supplementary Material 5 file. There were significant main effects of device [*F*(3, 1393) = 49,055.0, *p* < .001, *η*_G_^2^ = .61] and latency group [*F*(1, 1393) = 61,537.0, *p* < .001, *η*_G_^2^ = .97], as well as a significant interaction between device and latency group [*F*(3, 1393) = 62,230.0, *p* < .001, *η*_G_^2^ = .73]. Pair-wise comparisons revealed significant differences between all combinations of device and latency group (*p*s < .001). The interaction between device and latency group reflected that the difference between the lowest and highest latencies was smallest for the K-CL25, followed by the MT-PB and then the K-MX, and the largest difference was shown by the R-SD50. As is shown in Fig. 7a, the K-MX produced the lowest latency, followed by the R-SD50, then the K-CL25, and finally the MT-PB. One-sample two-tailed *t* tests against the test values 1 (representing 1 ms) revealed that the audio latencies for all devices and latency groups were significantly greater than 1 ms (*p*s < .001). This result fails to support the hypothesis that auditory feedback can be delivered by MIDI sound modules within 1 ms.

**Table 3:** Instruments with the lowest and highest latencies for each device

Type	Device	Latency Group	Instrument	Mean	*SD*	Min.	Max.
Sound module	MT-PB	Lowest	Xylophone	3.92	0.79	2.52	5.67
		Highest	ChoirAahs	13.61	2.82	1.95	19.27
	R-SD50	Lowest	RegHTom	2.54	0.04	2.47	2.65
		Highest	Reed organ	19.31	0.05	19.23	19.46
Keyboard	K-CL25	Lowest	Electric piano	3.04	0.21	2.61	3.76
		Highest	Harpsichord	11.23	0.18	10.91	11.90
	K-MX	Lowest	Tabla-tin	1.79	0.07	1.68	1.97
		Highest	Maracas-push	16.75	0.83	15.56	17.66

The latencies demonstrated by the harpsichord and vibraphone for each device are shown in Fig. 7b. Significant main effects of device [*F*(3, 1393) = 63340.0, *p* < .001, *η*_G_^2^ = .94] and instrument [*F*(1, 1393) = 39,555.0, *p* < .001, *η*_G_^2^ = .87] emerged, along with a significant interaction between device and latency group [*F*(3, 1393) = 62,302.0, *p* < .001, *η*_G_^2^ = .94]. Pair-wise comparisons revealed significant differences between devices for the xylophone (*p*s < .001), but the vibraphone only showed significant differences between the MT-PB and all other devices (*p*s < .001); no significant differences were evident between the K-CL25 and K-MX (*p* = *.*98), the K-CL25 and R-SD50 (*p* = *.*82), or the K-MX and R-SD50 (*p* = *.*22). Bayes factor *t* tests indicated extreme evidence for the null hypothesis for comparisons between the K-CL25 and K-MX (BF_H0_ = 98 ± 1.1%) and the K-CL25 and R-SD50 (BF_H0_ = 98 ± 1.3%). These results show partial support for the hypothesis that devices playing the vibraphone patch would have similar latencies. For the comparison between the K-MX and R-SD50, Bayes factor indicated extreme evidence for the alternative hypothesis (BF_HA_ > 1,000 ± 1.3%); in other words, there was no evidence that the null result could be accepted. This result contradicts the hypothesis that the devices playing the vibraphone patch would have similar latencies. The harpsichord produced significantly higher latencies than the vibraphone for three of the devices (MT-PB, R-SD50, K-CL25; *p*s < .001), but the reverse trend was found for the K-MX (*p* < .001). This result is interesting because it means that there are latency differences not only between devices, but also within devices, in how they generate MIDI audio. This point is further demonstrated by the larger disparity between the two instruments for the K-CL25 than for the other devices, including the MT-PB, which also uses GM. Overall, these results do not support the hypothesis that devices playing the same GM instrument would have similar latencies.

### Discussion

The results of Experiment 4 indicate that the latency of audio in response to a MIDI message varies not only between devices but also within devices. Moreover, none of the devices or instruments that were tested produced auditory feedback within 1 ms. It is also worth noting that the dedicated sound modules did not all produce lower latencies than the sound modules integrated into piano keyboards. In fact, the K-MX keyboard produced the lowest mean latency, then the R-SD50 sound module, then the K-CL25 keyboard, and finally the MT-PB sound module. This alternation indicates that integrated sound modules do not produce significantly higher latencies than dedicated sound modules and, in some cases (e.g., K-MX), produced lower latencies than dedicated sound modules. Regarding the GM standard, I observed evidence that different devices produce different latencies for the same instrument. Therefore, it is likely that the implementation of MIDI instruments varies from device to device, even under the GM standard.

## General discussion

In four experiments, I demonstrated how the SMIDIBT can be implemented to benchmark several types of MIDI devices that are used in each step of a response-to-feedback chain that uses MIDI. In testing these devices, each of the steps added latencies that were significantly greater than 1 ms and this was without adding the latencies of all devices within the chain to estimate the total latency that would be incurred. Therefore, the devices tested here indicate that 1 ms latencies are not achievable using commercially available MIDI setups. Of all the devices in the chain, it appeared that the percussion pad and/or sound module would add the largest proportion of the latency, depending on the device, sound module, and instrument. Sensorimotor synchronization studies that require low-latency and reliable timing for response collection and auditory feedback may need to employ other methods, such as analog signals running through digital acquisition cards (see Elliott, Welchman, & Wing, 2009) or options using microcontrollers (e.g., Tap Arduino; Schultz & van Vugt, 2016; see also Schubert, D’Ausilio, & Canto, 2013).

Regarding millisecond timing resolution, some have argued that temporal delays close to the order of 1.5 to 5 ms are perceivable and can be controlled by performers when producing music (Iyer, Bilmes, Wright, & Wessel, 1997; Moore, 1988). Earlier perceptual studies have provided evidence that humans can discriminate changes in temporal intervals of 1 ms (Lunney, 1974; Michon, 1964; Nordmark, 1968; but see Friberg & Sundberg, 1995, for a study that finds higher perceptual thresholds when using MIDI). Sensorimotor synchronization studies with period and phase perturbations have shown that interval changes of 10 ms can influence synchronization (e.g., Thaut, Miller, & Schauer, 1998), but the lower behavioral limit is unknown. Similarly, delayed auditory feedback research has shown that the negative mean asynchrony (i.e., the tendency for responses to precede the onsets of an isochronous pacing signal) increased as the delay increased, but again, the lower limit for delayed feedback to influence performance is unknown (50 ms was the smallest delay tested by Aschersleben & Prinz, 1997). It is an empirical question as to whether millisecond delays are perceived or affect performance; the acceptable levels of latency and variability for response collection and feedback generation are at the experimenter’s discretion. The SMIDIBT aims to help inform such decisions.

In the present study I used the SMIDIBT to benchmark several different MIDI devices and instruments but did not test all instruments exhaustively or comprehensively benchmark every MIDI device that has been used. This toolbox is provided so that experimenters can test their own latencies during the stimulus selection phase. Researchers with other MIDI devices who choose to adopt the SMIDIBT could make their latency measurements publicly available so that other researchers can also make an informed decision regarding which devices and instruments best suits their needs. Moreover, knowing these latencies could aid researchers in interpreting the results of sensorimotor synchronization experiments, particularly in the case of statistically nonsignificant results that might have arisen due to unwanted latencies or variability of response measurement and/or auditory feedback. Such data could be stored on the SMIDIBT website (https://band-lab.com/smidi_toolbox).

Another MIDI device that was not examined here is the MIDI piano keyboard used as a response recording device (e.g., Maidhof et al., 2014; Zamm et al., 2017). It is a methodological challenge to measure keyboard onsets because keys may have different travel times before they are triggered, so the FSR option used in the present study might not be suitable for keyboards. Moreover, in terms of ecological validity for nondigital pianos, the temporal delay between a key being struck to initiate the hammer hitting the string and the sound being received is unknown and would likely vary between different pianos and types of piano (e.g., grand, upright). Future experiments could use the SMIDIBT to benchmark piano keyboards using and FSR or other sensors, in order to measure key responses and the consequent MIDI and audio outputs.

### Conclusion

The schematics, scripts, and data from this study are available online to download for free (https://band-lab.com/smidib_toolbox), and any data submitted by other researchers will also be made available. The SMIDIBT is a means to benchmark MIDI devices so that experimenters can know whether or not undesired latencies and/or variability are introduced by these tools. It is then up to the experimenters to decide whether or not the latency and variability are acceptable for their purposes.

#### Author note

The author thanks Peter Pfordresher for donating the Sound Blaster Live PCI card and Roland SPD6 for this research, Sebastian Hertl for his aid with data collection, and Michael Schwartze and Rachel Brown for their comments on previous drafts of the manuscript. This research was funded by the author, who has no affiliation with any of the companies that developed the devices tested herein.

### Electronic supplementary material

ESM 1 (PDF 1722 kb)

ESM 2 (CSV 22 kb)

ESM 3 (CSV 22 kb)

ESM 4 (CSV 24 kb)

ESM 5 (CSV 57 kb)

## Appendix 2

**Table 4:** Send durations for the Arduino MIDI benchmark

Duration	Device	Method	Baud Rate	Mean	*SD*	Min	Max
Send	Due	Single byte	15,625	0.045	0.021	0.023	0.068
			31,250	0.045	0.021	0.023	0.068
			46,875	0.045	0.021	0.023	0.068
			62,500	0.045	0.021	0.023	0.068
		All bytes	15,625	0.045	0.021	0.023	0.068
			31,250	0.045	0.021	0.023	0.068
			46,875	0.045	0.021	0.023	0.068
			62,500	0.045	0.021	0.023	0.068
	Mega	Single byte	15,625	0.044	0.023	0.023	0.068
			31,250	0.046	0.023	0.023	0.068
			46,875	0.045	0.023	0.023	0.068
			62,500	0.047	0.023	0.023	0.068
		All bytes	15,625	0.047	0.023	0.023	0.068
			31,250	0.047	0.023	0.023	0.068
			46,875	0.047	0.023	0.023	0.068
			62,500	0.047	0.023	0.023	0.068

**Table 5:** Transit durations for the Arduino MIDI benchmark

Duration	Device	Method	Baud Rate	Mean	*SD*	Min	Max
Transit	Due	Single byte	15,625	0.605	0.042	0.499	0.680
			31,250	0.271	0.037	0.181	0.340
			46,875	0.165	0.034	0.091	0.227
			62,500	0.108	0.028	0.045	0.181
		All bytes	15,625	1.886	0.037	1.769	1.950
			31,250	0.919	0.032	0.816	0.975
			46,875	0.601	0.034	0.522	0.635
			62,500	0.439	0.033	0.363	0.476
	Mega	Single byte	15,625	0.603	0.032	0.567	0.658
			31,250	0.281	0.029	0.249	0.340
			46,875	0.176	0.028	0.136	0.249
			62,500	0.122	0.030	0.091	0.181
		All bytes	15,625	1.893	0.025	1.837	1.927
			31,250	0.938	0.024	0.884	0.975
			46,875	0.625	0.026	0.567	0.658
			62,500	0.462	0.023	0.408	0.499

**Table 6:** Read durations for the Arduino MIDI benchmark

Duration	Device	Method	Baud Rate	Mean	*SD*	Min	Max
Read	Due	Single byte	15,625	1.288	0.062	1.202	1.361
			31,250	0.663	0.047	0.544	0.726
			46,875	0.455	0.041	0.408	0.499
			62,500	0.352	0.037	0.249	0.408
		All bytes	15,625	0.059	0.025	0.023	0.091
			31,250	0.059	0.025	0.023	0.091
			46,875	0.060	0.025	0.023	0.091
			62,500	0.060	0.025	0.023	0.091
	Mega	Single byte	15,625	1.287	0.059	1.202	1.361
			31,250	0.663	0.054	0.522	0.726
			46,875	0.460	0.039	0.295	0.522
			62,500	0.352	0.040	0.204	0.408
		All bytes	15,625	0.056	0.025	0.023	0.091
			31,250	0.056	0.025	0.023	0.091
			46,875	0.056	0.025	0.023	0.091
			62,500	0.056	0.025	0.023	0.091

**Table 7:** Total durations for the Arduino MIDI benchmark

Duration	Device	Method	Baud Rate	Mean	*SD*	Min	Max
Total	Due	Single byte	15,625	1.939	0.047	1.837	2.041
			31,250	0.980	0.042	0.794	1.066
			46,875	0.666	0.048	0.590	0.726
			62,500	0.506	0.047	0.408	0.567
		All bytes	15,625	1.990	0.034	1.927	2.064
			31,250	1.024	0.029	0.975	1.088
			46,875	0.707	0.029	0.658	0.748
			62,500	0.544	0.029	0.499	0.590
	Mega	Single byte	15,625	1.934	0.055	1.859	2.018
			31,250	0.990	0.059	0.816	1.066
			46,875	0.681	0.037	0.544	0.748
			62,500	0.521	0.057	0.385	0.590
		All bytes	15,625	1.996	0.026	1.950	2.041
			31,250	1.041	0.028	0.998	1.088
			46,875	0.729	0.025	0.680	0.771
			62,500	0.565	0.028	0.522	0.612

## Appendix 3

**Table 8:** Descriptive statistics for intervals produced in the SMIDIBT route test in Experiment 2 for the baseline condition

Trigger	Interval	Mean Interval (ms)	*SD*	Min	Max
Send	1 ms	1.03	0.04	0.98	1.09
	2 ms	2.03	0.14	0.98	2.11
	3 ms	3.05	0.16	2.00	3.13
Sent	1 ms	1.03	0.01	1.02	1.04
	2 ms	2.03	0.13	1.02	2.06
	3 ms	3.05	0.16	2.04	3.08
Received	1 ms	1.03	0.05	0.95	1.11
	2 ms	2.03	0.14	0.95	2.13
	3 ms	3.05	0.16	1.97	3.15
Read	1 ms	1.03	0.04	0.91	1.11
	2 ms	2.03	0.14	0.93	2.18
	3 ms	3.05	0.16	1.97	3.15

**Table 9:** Descriptive statistics for latencies in the SMIDIBT route test in Experiment 2

Interval	Interface	Device	Mean	*SD*	Min	Max
1 ms	Baseline	None	0.288	0.040	0.227	0.317
	USB	LogiLink MIDI–USB	N/A	N/A	N/A	N/A
		M-Audio UNO	8.834	4.017	1.406	16.122
		Roland UM-ONE	2.358	0.324	1.519	3.401
	PCI	Labway Soundboard D66	1.639	0.380	1.179	3.084
		Sound Blaster Live	1.578	0.149	1.225	2.177
		TerraTec TT Solo 1-NL	1.783	0.481	1.179	3.107
2 ms	Baseline	None	0.269	0.036	0.227	0.317
	USB	LogiLink MIDI–USB	1.823	0.156	1.451	3.039
		M-Audio UNO	1.682	0.152	1.338	2.358
		Roland UM-ONE	2.337	0.325	1.519	3.424
	PCI	Labway Soundboard D66	1.475	0.146	1.202	2.086
		Sound Blaster Live	1.551	0.146	1.247	2.154
		TerraTec TT Solo 1-NL	1.475	0.146	1.202	2.086
3 ms	Baseline	None	0.264	0.032	0.227	0.317
	USB	LogiLink MIDI–USB	1.815	0.161	1.429	5.125
		M-Audio UNO	1.656	0.152	1.338	2.336
		Roland UM-ONE	2.324	0.325	1.519	3.333
	PCI	Labway Soundboard D66	1.469	0.145	1.202	2.086
		Sound Blaster Live	1.545	0.146	1.247	2.154
		TerraTec TT Solo 1-NL	1.469	0.145	1.202	2.086

## Footnotes

1 The actual intervals produced by the Arduino between consecutive triggers are shown in Appendix 3 (Table 8). The mean intervals corresponded with the intended intervals with 1.03, 2.03, and 3.05 ms produced for intervals 1, 2, and 3 ms, respectively.

2 The Focusrite mixer was used because it had a MIDI IN port, and originally I planned to record MIDI onsets using the MIDI IN of the mixer. However, I discovered that MIDI signals were recorded with different latencies between two different audio mixers and also between different brands of recording software. Thus, I decided only to report the data for the MIDI onsets obtained by the “read Arduino” of the SMIDIBT. A full test of audio mixers and audio recording software would be a valuable contribution to research that records MIDI signals using such hardware and software, but it is outside the scope of the present study. There is, however, no a priori reason to expect that audio mixers would differ in their ability to synchronize auditory signals.

3 Because several methods for onset detection exist, the raw data are available upon request. Here I measured the best approximation of the audio onsets, based on the voltages of the auditory signal. However, the subjectively perceived temporal loci of the onset—that is, the perceptual center (Marcus, 1981)—would likely differ between the audio patches. This avenue of research was beyond the scope of the present experiment.

4 The descriptive and inferential statistics for FSR-to-MIDI latencies in Supplementary Material 2 further support this proposition, with large differences between the ranges for the various instruments within devices. However, it would be impossible to know the precise source of these differences without first examining the software and code operating within each device.
