# Supplementary material for: The Schultz MIDI Benchmarking Toolbox for MIDI interfaces, percussion pads, and sound cards
Source: Behav Res Methods. 2018 Apr 17;51(1):204–34. doi: 10.3758/s13428-018-1042-7 (PMC6420415; doi:10.3758/s13428-018-1042-7)
Supplement: Supplementary file 1 — (PDF 1722 kb) [file 13428_2018_1042_MOESM1_ESM.pdf]

The Schultz MIDI Benchmarking Toolbox for MIDI interfaces, percussion pads, and sound cards

Reference Manual, Version 1.0, 2018

Benjamin G. Schultz

07 March 2018

Contents

1. Introduction 4
2. Software Installation 6
  2.1 Arduino Installation 6
    2.1.1 Find your serial communication port 7
    2.1.2 Uploading (flashing) code to the Arduino 7
  2.2 Arduino Serial Monitor 7
3. Hardware Configuration 9
  3.1 Force Sensitive Resistor (FSR) 9
    3.1.1 What to buy 9
    3.1.2 Setup and schematics 9
    3.1.3 Testing the FSR 12
  3.2 MIDI Send Arduino 13
    3.2.1 What to buy 15
    3.2.2 Setup and schematics 16
    3.2.3 Testing the Send Arduino 17
  3.3 MIDI Read Arduino 18
    3.3.1 What to buy 18
    3.3.2 Setup and schematics 19
    3.2.3 Testing the Read Arduino 22
4. MATLAB Code to extract onsets 23
  4.1 The SMIDIB Route Test 23
  4.2 The SMIDIB Percussion Pad Test 23
  4.3 The SMIDIB Sound Module Test 24
5. Troubleshooting 25
  5.1 Problems with the Arduino GUI 25
  5.2 Using the serial monitor 26
  5.3 The MATLAB Scripts Aren't Working! 26
  5.4 I'm having a problem you haven't thought of! 26
6. Citing this software 27
  6.1 I can't find your reference online! 27
  6.2 Contributions 27
7. References 28

Appendix A: List of components for Arduino 29
Appendix B: Electronics Vendors 30

1. Introduction

The Schultz MIDI Benchmarking Toolbox (SMIDIBT) is a package of C code and MATLAB scripts that can be used to test temporal latencies that occur when using MIDI devices to record responses and create auditory feedback using a typical MIDI setup (Figure 1a). Specifically, the SMIDIBT can measure the latencies of:

1. A MIDI signal being sent to a PC and sent out again (Figure 1b)
2. A MIDI signal after a MIDI percussion pad has been hit/tapped (Figure 1c)
3. Audio after a MIDI percussion pad has been hit/tapped (Figure 1c)
4. Audio after a MIDI signal has been sent (Figure 1d)

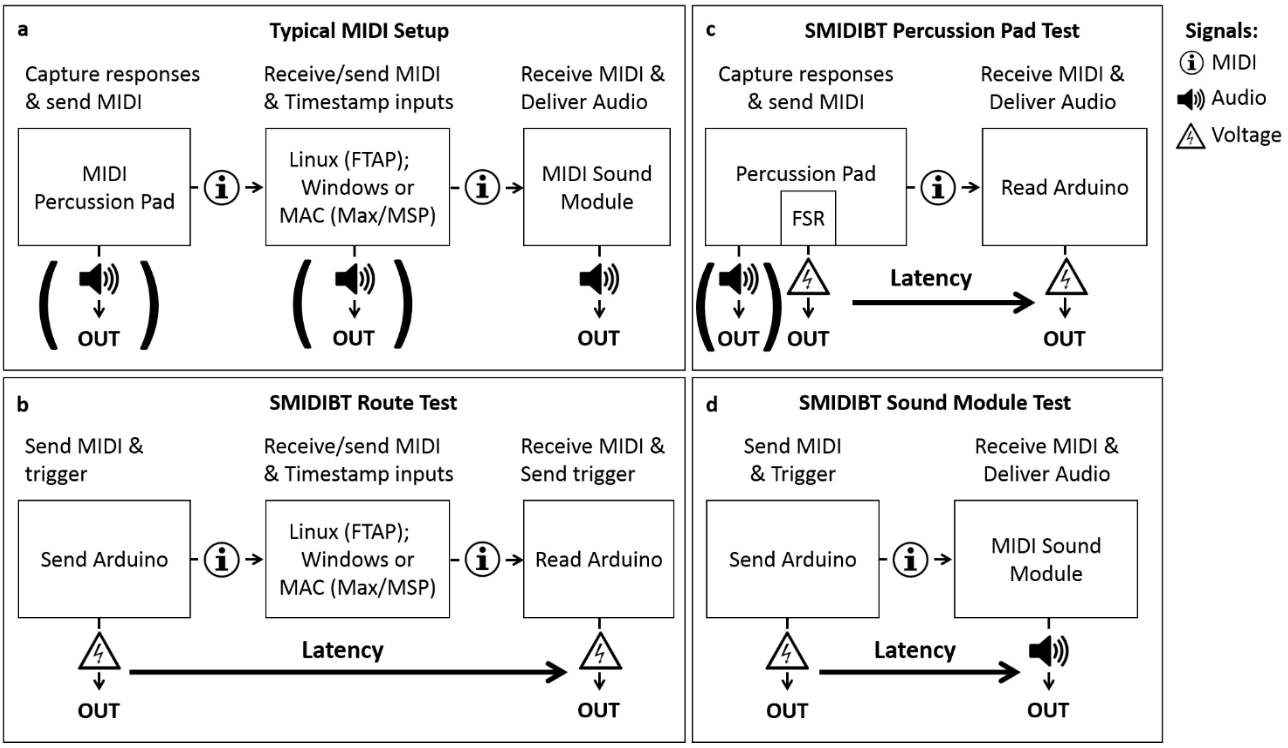

Figure 1. Layout of a typical MIDI setup (a) and the SMIDIBT setups that can be used to test the latencies of MIDI messages that are sent through computers and MIDI serial devices (b), the latencies of MIDI and audio from percussion pads (c), and the latencies of audio generated by MIDI sound modules (d). Each of the three components that can be sources of latency in a typical MIDI setup are represented in panels b (middle section of a typical MIDI setup), c (first section of a typical MIDI setup), and d (final section of a typical MIDI setup). The OUT audio and voltages can be compared to measure the veridical timing between inputs and outputs.

The SMIDIBT can be installed on any operating system (OS; Windows, Mac, and Linux) and does not put a heavy load on the CPU. The SMIDIBT uses the Arduino microcontroller, an open-source electronics platform that is easy to learn (Arduino,

2015). The Arduino can send and read MIDI signals and send accurate analog triggers to show when these signals are sent and received. These signals can be simultaneously recorded with audio to show the latency between the time a signal is sent and the time the audio is produced. The Arduino can also power a 5V force sensitive resistor (FSR) that can measure when a response occurs on a percussion pad or any other device that is triggered by force.

The temporal resolution of the SMIDIBT is limited by the sampling rate of the device used to record the Sent and Received triggers, and the audio output from the devices. Such devices include analog input boxes, oscilloscopes, and audio sound cards with at least two audio inputs.

The SMIDIBT software is provided at no charge (but without warranty) under the GNU Public License agreement (GPL v3, 2018).The GNU license allows you to use and modify the software in any way you like for “personal use”, but that any further distribution of the modified software must be done in source code form under the GNU license agreement. The SMIDIBT is available for unlimited use and modification, and can be downloaded from the repository:

http://band-lab.com/smidib_toolbox (Schultz, 2017).

If you have comments, bug reports, bug fixes, enhancement requests, or coded enhancements, please let me know so I can deal with them and/or choose to incorporate them into the standard version of the SMIDIBT. I would be happy to help you with any experiments you intend to perform, and please send me any scripts you use in your experiment that you think would help the community. Send comments and questions to benjamin.glenn.schultz@gmail.com.

Please cite Schultz (2018) in the write-up of any research you do that uses the SMIDIBT. I hope you enjoy using the toolbox as much as I have enjoyed putting it together!

2. Software Installation

This section explains how to install the software necessary to run the SMIDIBT. If you encounter any problems, please let me know the nature of the malfunction and the OS you are using. You will need to download the SMIDIBT from: http://band-lab.com/smidib_toolbox

2.1 Arduino Installation

Dedicated and very user-friendly software is available that allows you to upload code to Arduino, called the Arduino IDE (Integrated Development Environment). You can download the Arduino IDE from here: http://arduino.cc/en/Main/Software by selecting your OS, then following the instructions. This is the software you will use when you upload the Arduino codes (and, eventually, your own) onto the Arduino. Once the Arduino software is installed you will be able to test some of the basic functions of the Arduino outlined in sections 3.1 and 3.2.

In some older versions of Linux (e.g., Ubuntu 12.04), the Arduino IDE might be installed incorrectly or install an older version of the Arduino IDE. There are two solutions: 1) upgrade to a more recent version of Ubuntu (e.g., 14.04 or later), or 2) download the executable file. To download the executable file, go the Arduino webpage (www.Arduino.cc), go to Download, and download the Linux archive (32 bits or 64 bits depending on your system). This will give you a .tar.xz file to download (something like arduino-1.6.1-linux32.tar.xz). Put it somewhere where you can find it, then go there with the file manager, and extract the archive by right-clicking on it and selecting "Extract here". This should create a new folder in the same place, called something like arduino-1.6.1. Inside, there should be an executable file called "arduino". Double click on it, and you should be prompted whether you want to run "arduino' or display its contents: select "Run".

If you are using Linux, the first time you run the Arduino IDE it may ask you to join the dialout group. Click “Add” and restart your computer for these settings to take effect. What this does is ensure that your user account has permissions to read from serial communication ports so your computer can communicate with the Arduino.

In case you want to learn more about Arduino and developing code for it, please refer to the excellent tutorials on http://arduino.cc/en/Tutorial/HomePage. However, such detailed knowledge is not required if you want to use the existing scripts.

2.1.1 Find your serial communication port

The Arduino device will communicate with your computer through a USB cable. Once connected, your operating system (OS; e.g., Windows, Linux, Mac) will set up a so-called *serial communication port* that connects to Arduino. You need to find out which port this is. In Windows (XP, 7, and 8/8.1) you can check your device manager and look for your Arduino device. It should show "(COM#)" under the device, where "#" is the number of the serial communication port (called COM port in Windows) the device is using. If you are using a Mac or Linux, you can type the following into the terminal: *ls /dev/tty.**

This will produce a list of all serial ports. By comparing this list before and after connecting your USB cable, you can find which serial port newly appeared when Arduino connected, and that will be your designated port. If you are not using other devices, the port that is assigned by default is */dev/ttyACM0*

Optionally, you can instruct Mac or Linux to always map the Arduino to a particular port, by writing an "*udev*" rule. Please refer to an "*udev*" tutorial for this option, since it is beyond the scope of this manual.

2.1.2 Uploading (flashing) code to the Arduino

To benchmark MIDI devices with the Arduino, you will need to upload the SMIDIBT codes to the Arduino. Open up the Arduino IDE. Uploading code to the Arduino is done by opening the code you want to upload, connecting your Arduino through USB, and clicking on the upload icon (a right arrow) or by going to "File" and clicking "Upload". If the code is uploaded correctly, you will receive some white text in the black box at the bottom of the Arduino IDE. If something goes wrong, you will receive some orange text that will give clues as to what the error is (see section 4.2). Please ensure that the code is uploaded to the correct Arduino (and on the correct Serial communication port or COM port).

2.2 Arduino Serial Monitor

To open the serial monitor, go to Tools → Serial Monitor, or press Ctrl + Shift +M simultaneously (see Figure 2).

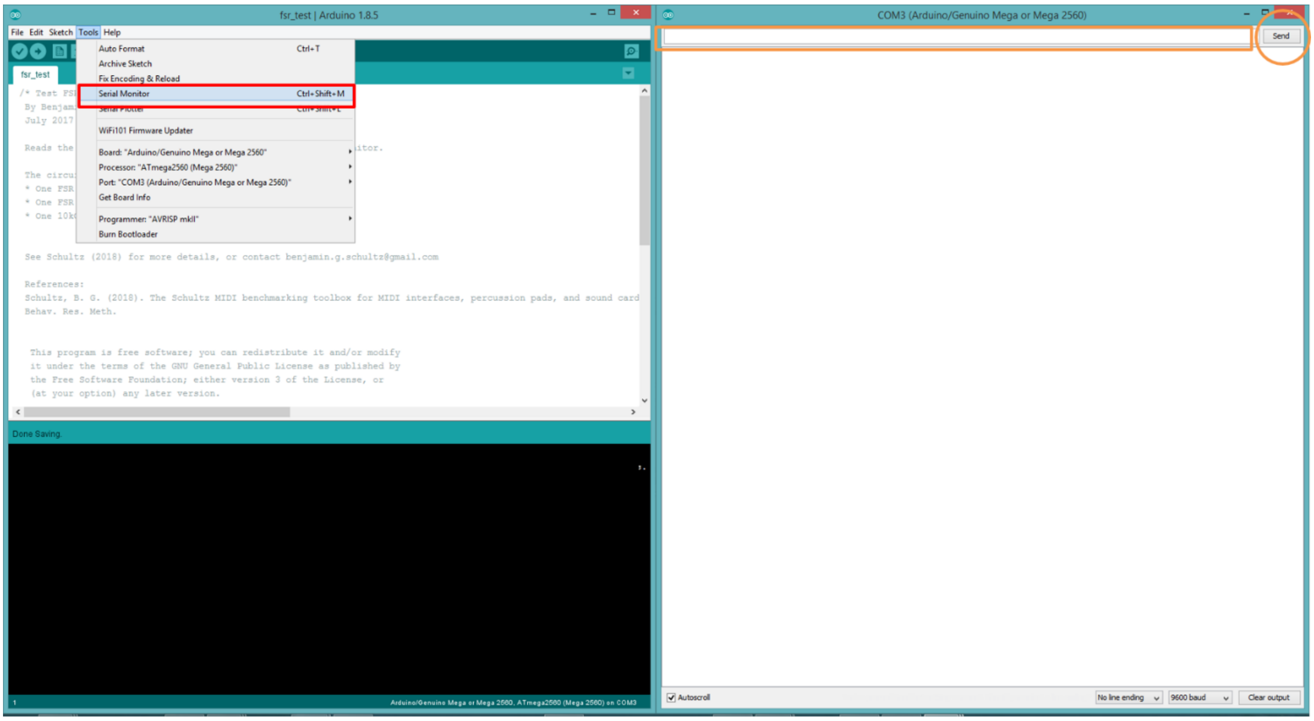

Figure 2. Arduino interface (left panel) and serial monitor (right panel). The red rectangle show the command to open the serial monitor. The orange rectangle shows where commands can be sent to the Arduino to start "Send" tests. The orange circle shows the button you can press to send the command to start the test (or you can press enter after typing).

3. Hardware Configuration

Here, I give you step-by-step instructions for how to build the Arduino, connect the sensors, MIDI outputs, and audio outputs, and test the equipment to make sure everything works as we go along. This should help with trouble shooting later. As a general rule, the colouring of my wires will consistently follow this convention: red is connected to power pins (3.3V or 5V), black is connected to ground pins (GND), green is connected to the MIDI serial inputs (RX3, pin 14) and outputs (TX3, pin 15) purple is connected to analog inputs for the FSR (A0 to A5), and blue is connected to audio outputs (pulse-width modulation pins 9, 10, or 11) used as the MIDI triggers.

3.1 Force Sensitive Resistor (FSR)

This section describes how to connect the force sensitive resistor (FSR) to the Arduino and record tap onsets for MIDI percussion pads. If you don't need to test MIDI percussion pads, then you can skip to section 3.2.

3.1.1 What to buy

For the FSR setup, you will need to purchase an Arduino UNO, an FSR (I use a square one in this example), a breadboard, some hook up wire, a 2-pin 3.5mm screw terminal (optional), a headphone jack (I use a 3.5mm TRRS jack breakout board), and a 10kΩ resistor with the colours brown, black, orange, and gold (see Appendix Y). The FSR is quite fragile, so I describe an alternative to soldering wires to the FSR (see https://learn.adafruit.com/force-sensitive-resistor-fsr/connecting-to-an-fsr), specifically, connecting the FSR directly to the breadboard (see Figures 3 and 4a) by skipping the optional steps (1-3). If you do not have much (or any) experience with soldering, I recommend you either use the described method, or use screw terminals or clamp connectors (i.e., alligator clips). I chose the TRRS jack with the breakout board because it is easier to connect the headphone to the breadboard, and to know to what the connections refer (i.e., TIP, RING1, RING2, and SLEEVE; see Figures 3 and 4a and 4b):
https://www.sparkfun.com/products/11570

3.1.2 Setup and schematics

While the Arduino is OFF (the power and USB are not connected), arrange the cables as follows (see Figure 3):

1. (Optional step) *Carefully(!)* solder the end of the red hook up wire to one side of the FSR (or screw terminal).
2. (Optional step) *Carefully(!)* solder the end of the purple hook up wire to the other side of the FSR (or screw terminal).

3. (Optional step) If using a screw terminal, insert the two FSR pins into the two pin holders and use a small screwdriver to screw the pins in place.
4. Connect a red wire between the breadboard row containing the left pin of the FSR and the Arduino 5V pin (or other end of the red wire connected to the FSR pin to the Arduino's 5V pin).
5. Connect a purple wire from the same breadboard row as the right FSR pin (or purple wire connected to the FSR) to the Analogue 0 pin (A0) of the Arduino.
6. Connect a third purple wire from the same row of the breadboard as the first purple wire to the "TIP" ring of the audio jack.
7. Connect a black wire from the Arduino's ground (GND) pin to "RING2" and/or "SLEEVE" (depending on your audio connection).
8. The 10kΩ resistor acts as a voltage divider between the ground pin and the incoming signal from the FSR. Connect one end of the resistor to the same breadboard row as the purple wires, and the other end to a different breadboard row.
9. Connect one end of a second black wire to the same breadboard row as the resistor (but NOT the same row as the purple wires), and the other end of the black wire to the Arduino's ground (GND) pin.

Congratulations! You have just connected the FSR to the Arduino. Now we should test that everything works.

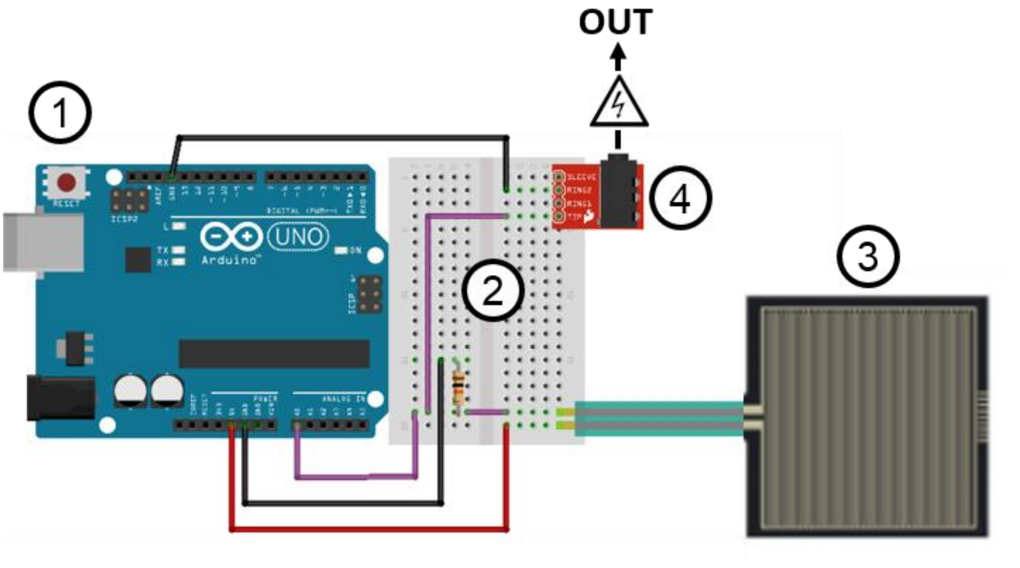

Figure 3. Schematic of the wiring for the force sensitive resistor (FSR) with audio jack connection for the Arduino. Labels refer to the following: 1. Arduino UNO, 2. Breadboard, 3. FSR, 4. a TRRS 3.5mm headphone jack. A 10kΩ resistor is used on the breadboard. See text for construction instructions. Note that the purple wires are orange in Figure 4a. Figure produced using fritzing (Knörig, Wettach, & Cohen, 2009).

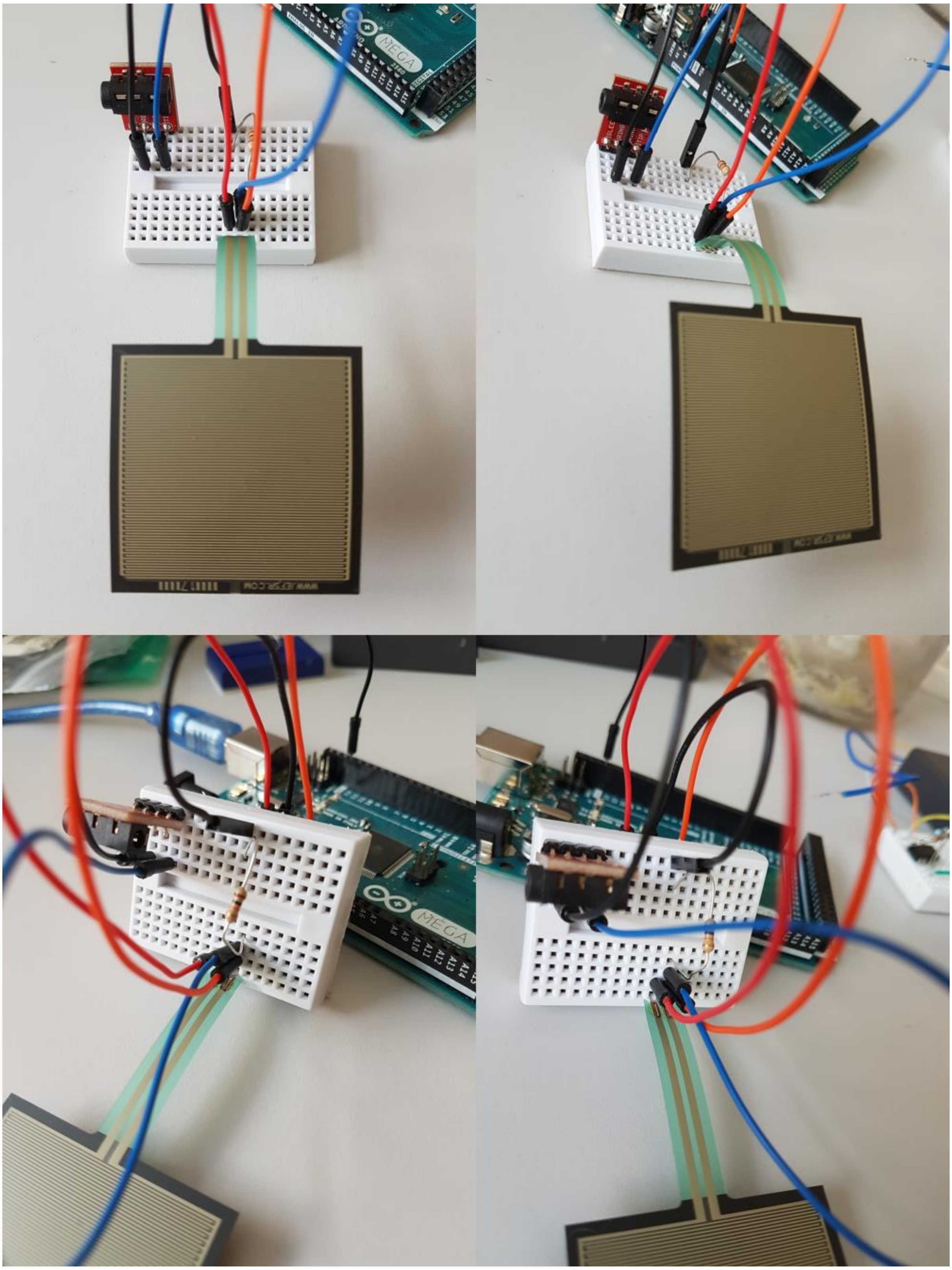

Figure 4a. Photos of the FSR setup without soldering. For those with trouble identifying colour, the left cable is red and the right cable is orange. See Figure 3 for instructions.

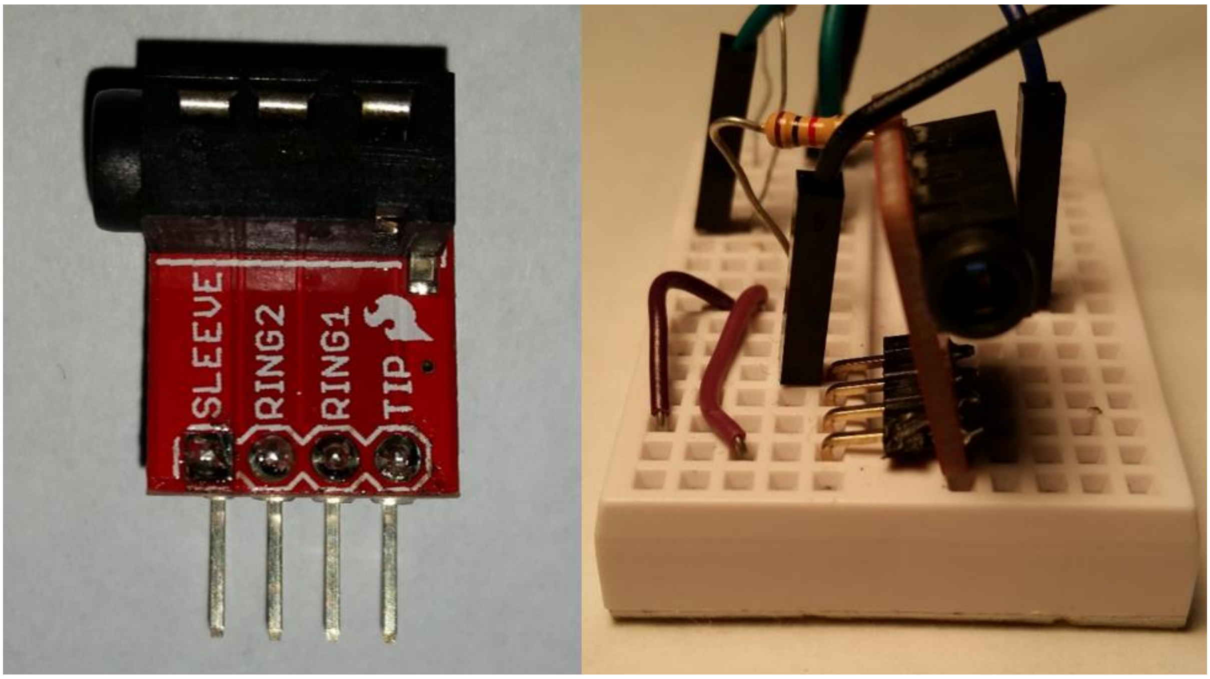

Figure 4b. Photos of the headphone jack (left panel) and breakout board (right panel) that connects to the FSR and ground (GND pin). See Figure 3 for instructions.

3.1.3 Testing the FSR

Once you have connected the FSR to the Arduino, we can test that the FSR is working. Plug the USB cable into the Arduino (with the other end connected to your computer). The lights on the Arduino should come on, indicating that you are now connected and the power is on (the Arduino is USB powered). If the lights do not come on, then your USB cable might need replacing or the Arduino you purchased may have shuffled off this mortal coil (i.e., died) and you will need to speak to your retailer. If the lights go on, then we may proceed.

Open the “fsr_test.ino” sketch using the Arduino software. Upload the sketch as shown in section 2.1.2 and open the “Serial Monitor” as shown in section 2.2. You should see something like the output in Figure 5. Try tapping on the FSR and see what it looks like. This is how continuous data is read into the Arduino from the FSR. Once you have connected the audio jack of the SMIDIBT FSR setup to a sound card (or other similar device, e.g., an oscilloscope or analog input box) then you have all you need to start testing the audio latencies of MIDI percussion pads. Check that, when you tap, the signal from the FSR is being read through the audio

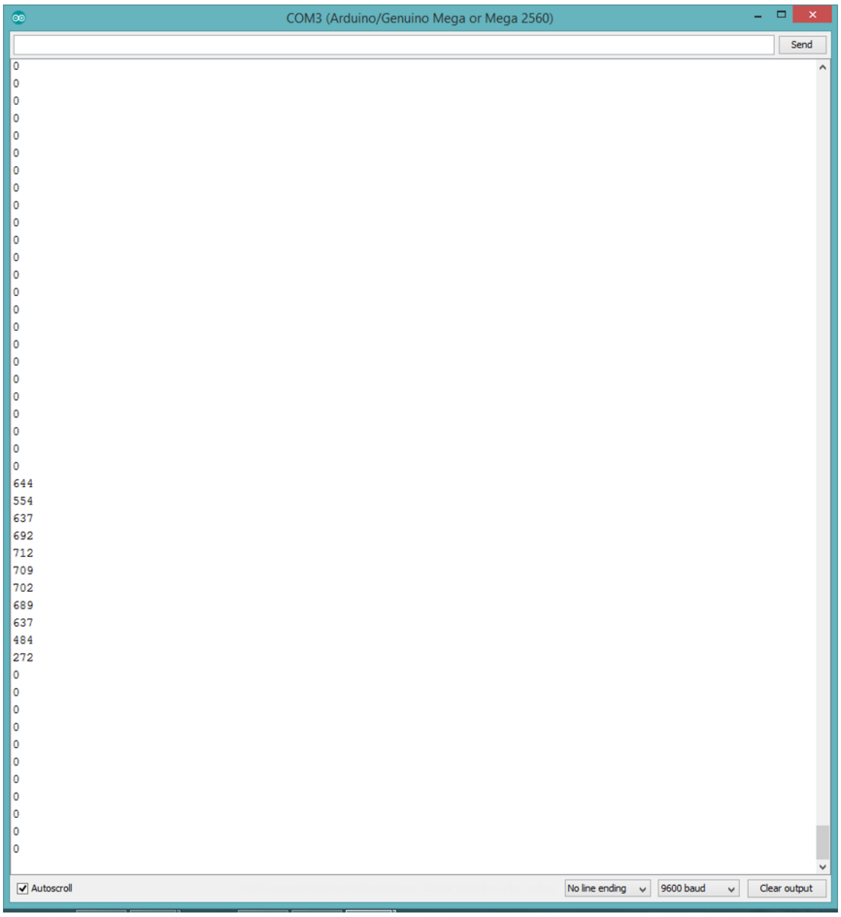

Figure 5. Serial monitor showing a single tap from the FSR attached to the Arduino. Numbers greater than zero represent a response and the response ends when the numbers return to zero.

3.2 MIDI Send Arduino

Sending MIDI messages from the Arduino can be performed without any soldering using the schematic in Figure 6a (see also Figure 7). This section describes how to send MIDI messages and connect the pulse-width modulation (PWM) pin to the headphone jack to produced triggers before and after the MIDI message is sent.

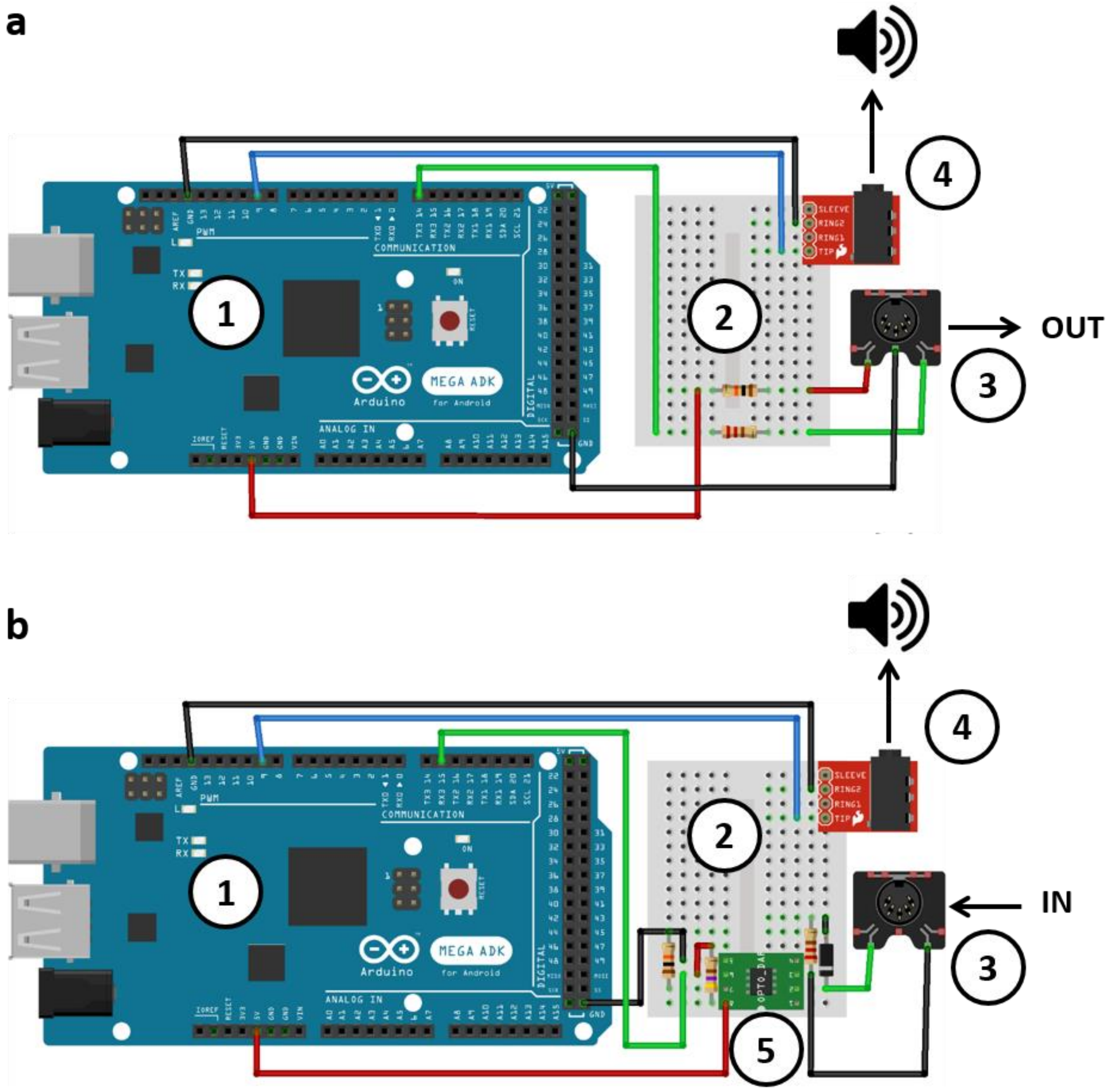

Figure 6. Schematic of wiring diagram for the Schultz MIDI benchmarking toolbox setup. Component numbers correspond to: 1. Arduino Mega, 2. Breadboard, 3. MIDI connector, 4. Audio jack, and 5. Optocoupler (6N138). Electric wires are indicated by black (grounds), red (power), green (MIDI signals), and blue (Audio signals) wires. The top panel (a) shows the “send Arduino”. The green wire is connected to a 220Ω resistor and the red wire is connected to a 10kΩ resistor. If using an Arduino Due, connect the red wire to the 3.3V pin instead of the 5V pin. The bottom panel (b) shows the “read Arduino”. The resistors from left to right are 10kΩ, 470Ω, and 220Ω. Note the direction of the black diode (1N4148) on the far right as it is unidirectional. This wiring diagram will allow prospective users to precisely reproduce the set up from the hardware components. Figure created using fritzing (Knörig, Wettach, & Cohen, 2009).

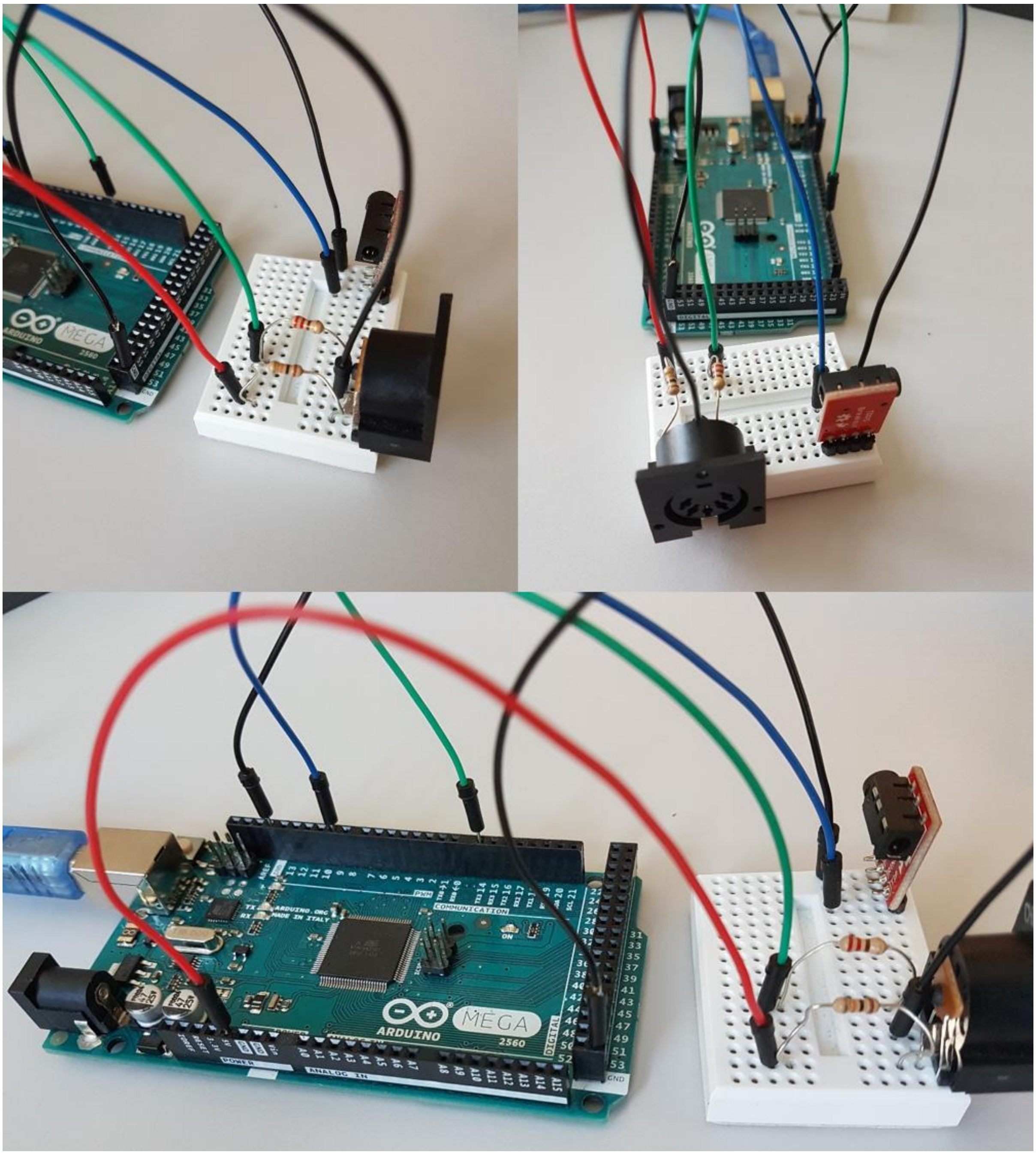

Figure 7. Photographs of the SMIDIBT Send Arduino connected without any soldering (assuming your audio jack came with male header pins).

3.2.1 What to buy

For the SMIDIBT Send Arduino, you will need to purchase an Arduino MEGA (or Due), a headphone jack (I use a 3.5mm TRRS jack breakout board, see Figure 4b), male headers (4 pins), a breadboard, some hook up wire, a female MIDI connector, and one 220Ω resistor (red, red, brown, gold) and one 10kΩ resistor (brown, black, orange, gold) (see Appendix A). I chose the Sparkfun female MIDI connector (PRT-

09536) because the spacing is more breadboard friendly than other models and, therefore, requires no soldering for the purposes of the SMIDIBT.

3.2.2 Setup and schematics

While the Arduino is OFF (the power and USB are not connected), arrange the cables as follows (see Figure 6a):

1. Insert the female MIDI connector to the breadboard ensuring that each of the five pins is in different rows and that there is room leftover to access the other breadboard pins of those rows (see Figure 7). Note that some MIDI connectors have two pins at the very front that are used for stability but are, otherwise, not used.
2. The MIDI connector has five inputs pins that correspond to the five pins on the back, connected sequentially (see Figure 8). Looking from the back, connect the 220Ω resistor to the middle-left pin (pin 4 in Figure 8) and then to an empty row on the breadboard.
3. Connect one side of a green wire to the empty row containing the other end of the 220Ω resistor, and the other side to the TX3 pin (pin 14) on the Arduino.
4. Looking from the back, connect a black wire to the middle pin (pin 2 in Figure 8) and to a ground (GND) pin on the Arduino.
5. Looking from the back, connect the 10kΩ resistor to the middle-right pin (pin 5 in Figure 8) and then to an empty row on the breadboard.
6. Connect one side of a red wire to the empty row containing the other end of the 10kΩ resistor, and the other side to the 5V pin on the Arduino MEGA, or the 3.3V pin on the Arduino Due.
7. If male headers are not supplied for the headphone breakout board, solder the short side of the male header pins to the four pins of the headphone breakout board (labelled TIP, RING1, RING2, and SLEEVE). Once cooled, insert the long end of the male header pins into the breadboard, ensuring that each pin is in a different 5-hole row.
8. Connect one end of a blue wire to the Arduino’s digital pin 9 (~9), and the other end to the row connected to the TIP ring of the audio breakout board.
9. Connect one end of the black wire to the row containing RING2 and the other end to a ground (GND) pin of the Arduino.

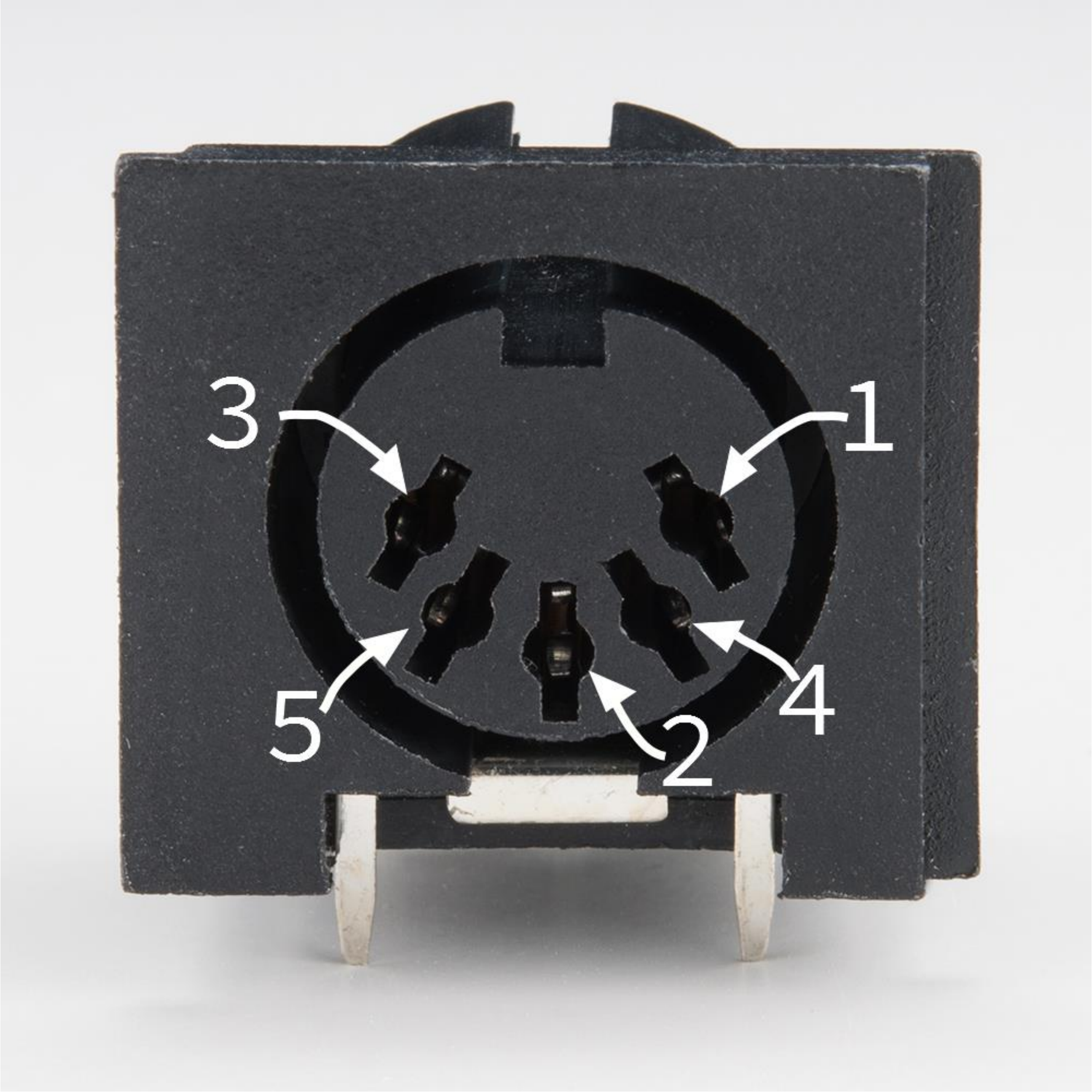

Figure 8. Pin labels for the female MIDI connector. Note that the pins on the back refer to the same order but mirrored (i.e., the back pins from left to right are 1, 4, 2, 5, 3). Only pins 2, 4, and 5 are used here.

3.2.3 Testing the Send Arduino

First, upload the SMIDIBT_Send.ino sketch using the method shown in Sections 2.1.1 and 2.1.2. Then open the Serial Monitor as shown in Section 2.2. When you send anything from the command window, you should receive some text showing that the benchmark is starting. The light emitting diode (LED) on the Arduino next to "TX" should also light up each time a message is sent. Moreover, if the audio jack is connected to a sound card or mixer, it should receive the triggers each time a message is sent. Finally, if the MIDI is connected to a device, one 3-byte MIDI message per trigger will be received.

3.3 MIDI Read Arduino

Reading MIDI messages with the Arduino can also be performed without any soldering using the schematic in Figure 6b (see also Figure 9). This section describes how to read MIDI messages and connect the PWM pin to the headphone jack to produced triggers when a MIDI message is received and read.

3.3.1 What to buy

For the SMIDIBT Read Arduino, you will need to purchase an Arduino MEGA (or Due), a headphone jack (I use a 3.5mm TRRS jack breakout board, see Figure 4b), male headers (4 pins), a breadboard, some hook up wire, a female MIDI connector, an optocoupler (6N138), a diode (1N4148), one 220Ω resistor (red, red, brown, gold), one 10kΩ resistor (brown, black, orange, gold), and one 470Ω resistor (yellow, violet, brown, gold) (see Appendix A). Again, I chose the Sparkfun female MIDI connector (PRT-09536) because the spacing is more breadboard friendly than other models and, therefore, requires no soldering for the purposes of the SMIDIBT. Similarly, the chosen optocoupler can be placed on a breadboard so the top and bottom rows can be separated by the middle of the breadboard (see Figures 6b, 9, and 10). Unlike resistors, the direction of the diode in this circuit is important because the current can only go in one direction (usually from the largest unmarked area towards the thickest line, but check with the supplier; see Figure 11).

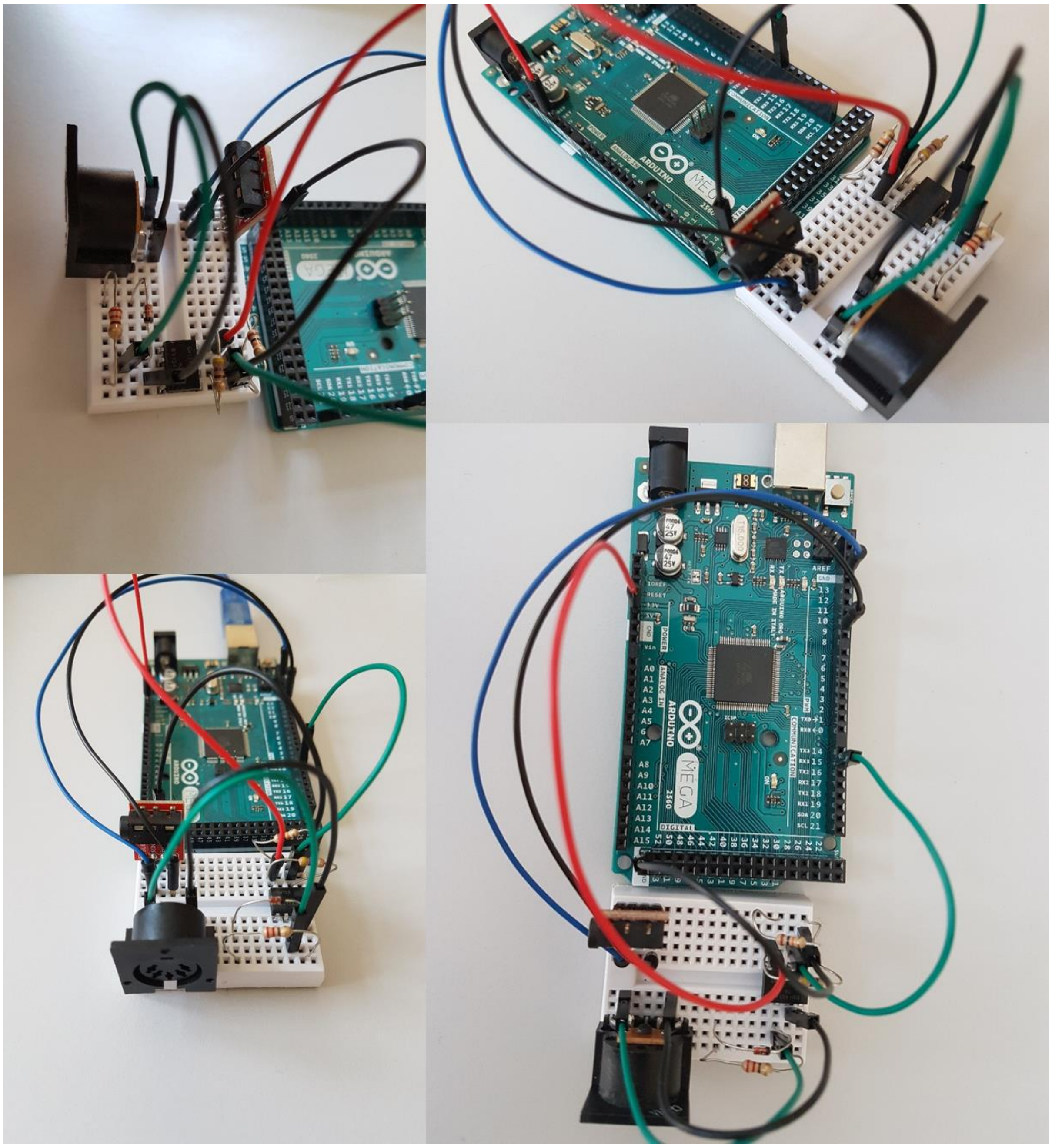

Figure 9. Photographs of the SMIDIBT Read Arduino connected without any soldering (assuming your audio jack came with male header pins).

3.3.2 Setup and schematics

While the Arduino is OFF (the power and USB are not connected), arrange the cables as follows (see Figure 6a):

1. Insert the female MIDI connector to the breadboard ensuring that each of the five pins is in different rows and that there is room leftover to access the other breadboard pins of those rows (see Figure 9). Note that some MIDI

connectors have two pins at the very front that are used for stability but are, otherwise, not used.

2. Connect the optocoupler so each pin of the bottom pins (pins 1-4) is in a separate row on the same half of the breadboard as the female MIDI connector (see Figures 9 and 10). Each pin of the top pins (pins 5-8) of the optocoupler should be in a separate row on the opposite half of the breadboard.
3. Connect one side of a green wire to the breadboard row containing MIDI pin 5 and the other end to the breadboard row containing optocoupler pin 2.
4. Connect one side of a black wire to the breadboard row containing MIDI pin 4 and the other end to the breadboard row containing optocoupler pin 3.
5. Connect one end of the 220Ω resistor (red, red, brown, gold) to the breadboard row containing optocoupler pin 3, and the other end to an empty breadboard row.
6. Connect the diode from the previously empty breadboard row that now contains the 220Ω resistor to the breadboard row containing optocoupler pin 2. The anode side should be in the same row as the 220Ω resistor and the cathode side should be in the same row as optocoupler pin 3. This completes the input side of the breadboard as shown in the bottom right panel of Figure 9.
7. Connect the 470Ω resistor to the breadboard rows containing optocoupler pins 8 and 6.
8. Connect the 10kΩ resistor to the breadboard rows containing optocoupler pins 7 and 5.
9. Connect one side of a red wire to the breadboard row containing optocoupler pin 8 and the other end to the 5V pin on the Arduino.
10. Connect one side of a green wire to the breadboard row containing optocoupler pin 6 and the other end to the RX3 pin (pin 15) on the Arduino.
11. Connect one side of a black wire to the breadboard row containing optocoupler pin 5 and the other end to the GND pin on the Arduino.
12. If male headers are not supplied for the headphone breakout board, solder the short side of the male header pins to the four pins of the headphone breakout board (labelled TIP, RING1, RING2, and SLEEVE). Once cooled, insert the long end of the male header pins into the breadboard, ensuring that each pin is in a different 5-hole row.
13. Connect one end of a blue wire to the Arduino's digital pin 9 (~9), and the other end to the row connected to the TIP ring of the audio breakout board.
14. Connect one end of the black wire to the row containing RING2 and the other end to a ground (GND) pin of the Arduino.

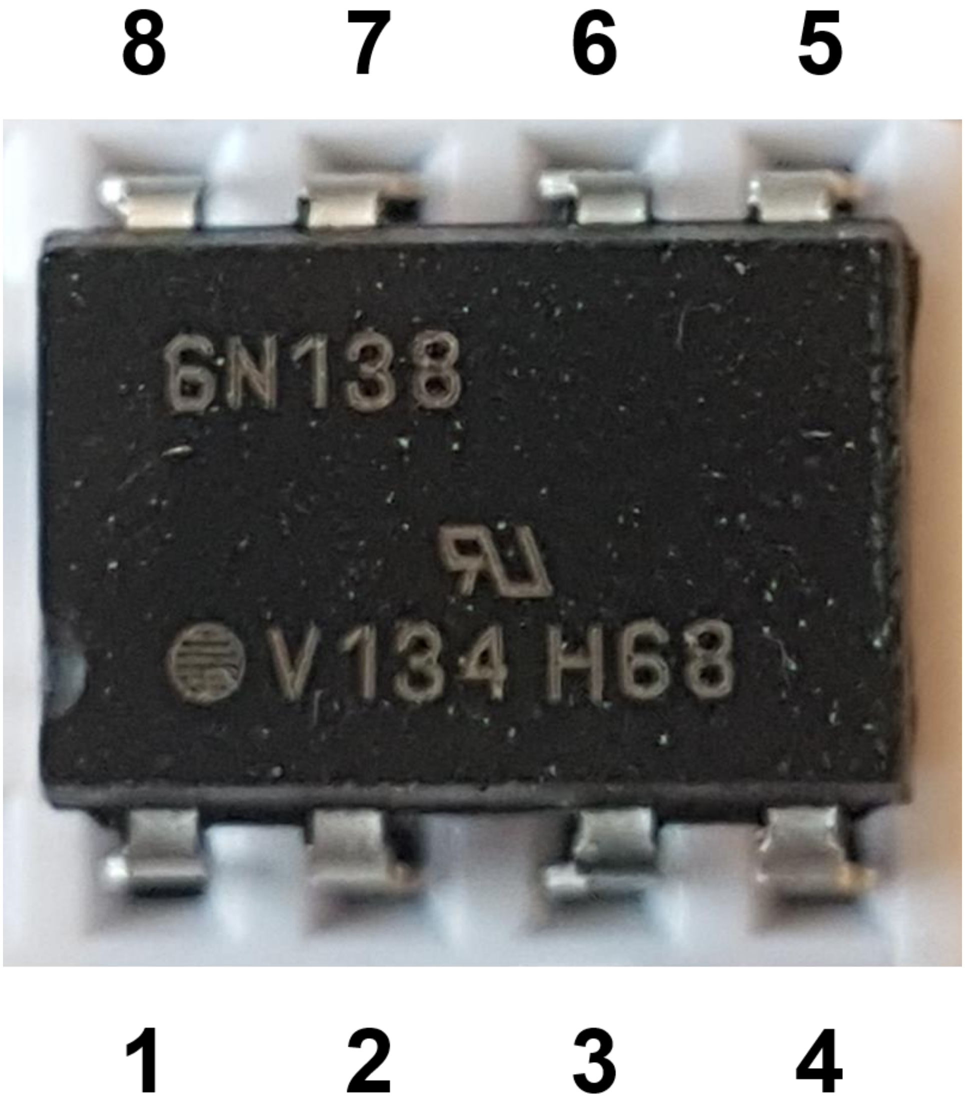

Figure 10. Pin labels for the optocoupler (6N138). The bottom row (1-4) represents the inputs (i.e., where the MIDI message will first enter) and the top row (5-8) represents the outputs (i.e., where the MIDI message will leave before going to the Arduino for processing).

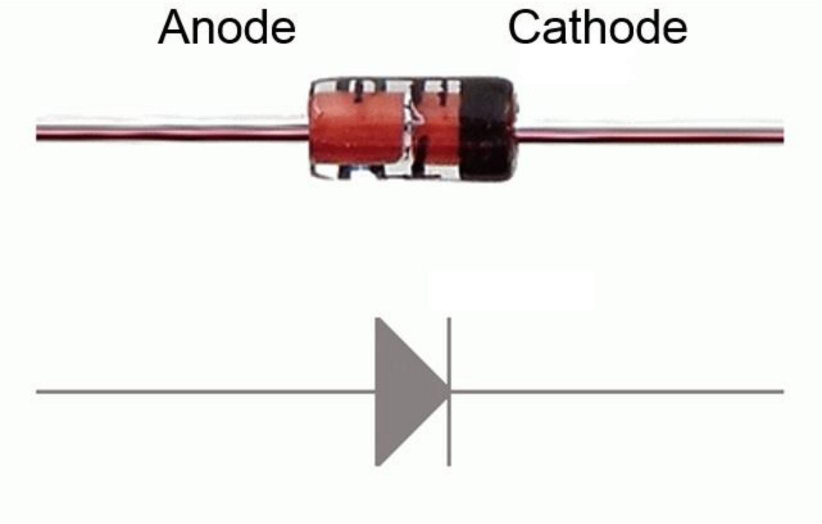

Figure 11. Directionality of the diode (1N4148) used for the Read Arduino. The voltage goes from the Anode (left) to the Cathode (right).

3.3.3 Testing the Read Arduino

First, upload the SMIDIBT_Read_Single.ino sketch (as this was a faster read method than the *3bytes method) using the procedure shown in Sections 2.1.1 and 2.1.2. Whenever the Read Arduino receives a MIDI message, a trigger will be sent to the audio card. The light emitting diode (LED) on the Arduino next to “RX” should also light up each time a message is received. You are now ready to start testing your MIDI devices!

4. MATLAB Code to extract onsets

The MATLAB scripts already contain documentation within each script but I briefly describe the scripts here. There were three types of tests performed in Schultz (2018): The route test (Experiments 1 and 2), the percussion pad test (Experiment 3), and the sound module test (Experiment 4). The scripts associated with each of these tests are described in the following sections

4.1 The SMIDIBT Route Test

This test examines the time it takes for a MIDI message to be sent and received (see Figure 1b). The MIDI message could either go directly from the Send Arduino to a Read Arduino, or go through another MIDI reading and writing device (e.g., through USB-MIDI and through a computer). The two audio files that recorded the triggers from the Send Arduino and Read Arduino should be placed in the same folder ("route_data"), and the following naming system should be implemented:

A_B_C_D_E_F.wav

Where A is the MIDI-USB or MIDI-PCI device, B is the software used to read and write the MIDI data on the computer, C is the type of test (for now, only a dummy variables that reflects the route test), D is the trial number, E is the interval between MIDI sends, and F is whether the audio file is for the Send ("send") or Read ("read") Arduino. Each of these must be separated by a single underscore "_".

Running the script "SMIDIBT_route_latencies.m" will return an excel spreadsheet of the latencies between consecutive MIDI events. In other words, you will receive the duration of sending a MIDI message, the duration of reading a MIDI message, the duration between when a MIDI message is sent and received (i.e., transit duration), and the total duration from when a MIDI message starts to be sent until it is fully received and read. The onsets and offsets for each file pair are calculated in "SMIDIBT_route_onsets.m".

4.2 The SMIDIBT Percussion Pad Test

This test examines the time it takes for a MIDI percussion pad to send a MIDI message and produce audio after being struck (see Figure 1c). Three audio files are expected: 1) the voltages (i.e., resistance) from the force sensitive resistor), 2) the triggers from the Read Arduino, and 3) the audio from the percussion pad (if any). All audio files should be placed in the same folder ("percussionpad_data"), and the following naming system should be implemented:

A_B_C_D.wav

Where A is the percussion pad device name (or abbreviate thereof), B is the MIDI sound bank used to produce the audio, C is the trial number, and D is whether the audio file is for the FSR ("fsr"), triggers from the Read Arduino ("trigs"), or percussion pad audio ("audio"). Each of these must be separated by a single underscore "_".

Running the script "SMIDIBT_percussionpad_latencies.m" will return an excel spreadsheet of the latencies between the FSR readings (indicating the time the percussion pad was struck), the Read Arduino trigger onsets and offsets, and the percussion pad audio onsets. The onset and/or offset times for the files are calculated in "SMIDIBT_percussionpad_onsets.m".

4.3 The SMIDIBT Sound Module Test

This test examines the time it takes for a MIDI sound module to receive a MIDI message and produce audio (see Figure 1d). Two audio files are expected: 1) the triggers from the Read Arduino and 2) the audio from the sound module. All audio files should be placed in the same folder ("soundmod_data"), and the following naming system should be implemented:

A_B_C.wav

Where A is the sound module device name (or abbreviate thereof), B is the MIDI sound bank used to produce the audio, and C is whether the audio file is for the Send Arduino triggers ("trigs") or the sound module audio ("audio"). Note that "Trial" was removed for the sound module because you can simply set the Send Arduino script to send a set number of MIDI messages at a regular interval instead of collecting multiple trials between which human interference is required (in other words, these tests took a LONG time to perform, especially for sound banks with slow envelope decay). Each of these must be separated by a single underscore "_".

Running the script "SMIDIBT_soundmod_latencies.m" will return an excel spreadsheet of the latencies between the Send Arduino trigger onsets and offsets, and the sound module audio onsets. The onset and/or offset times for the files are calculated in "SMIDIBT_soundmod_onsets.m".

5. Troubleshooting

Anything that can go wrong, will go wrong (*Murphy's Law*). I know from experience that things won't always go smoothly. Here I have tried to pre-empt issues that may arise and provide the quickest way to find and fix the problem.

5.1 Problems with the Arduino GUI

I haven't had any problems with the Arduino GUI yet, but some issues are bound to come up at some point. Here are the usual suspects and solutions for problems you may encounter with the interface.

1. Is it plugged in? Are the Arduino lights on?
   If the Arduino lights are not on, then there is either something wrong with the USB cable or the Arduino might be broken (or "bricked"). These two problems can only be solved by replacing the cable or Arduino.
2. Is the USB being recognized?
   If the Arduino USB is connected through a secondary source (e.g., a keyboard or a monitor) then it might not be read correctly by the computer as a serial port. Ensure that the USB is directly connected to a computer USB port. This issue will affect the uploading of Arduino code and the reading of data through the USB.
3. Is the wiring correct?
   Double-check (and triple-check!) that the wires are in the correct pins and rows, and that no wires are connected that shouldn't be. See throughout Section 3 for examples of the wiring and connections.
4. Do you have the correct serial communication port number?
   It is possible that you have selected the wrong serial communication (COM) port number when uploading your code or sending a command. Check again by following the instructions in section 2.1.1.
5. Did the Arduino code upload correctly?
   Follow the instructions in section 2.1.2. Check the black display in the Arduino GUI when you upload your script. If there is any orange text, then something went wrong. I suggest that you go back to the source code to make sure that no errors have been introduced. If you are still receiving errors (i.e., orange text), ensure that you have the correct libraries imported and that your variables have been defined.
6. If you have gone through all of these steps and you are still having issues, please email me with the specifics of the problem, with error messages and system information (e.g., operating system):
   benjamin.glenn.schultz@gmail.com

5.2 Using the serial monitor

The Arduino serial monitor can also be used to test the output of the Arduino if you are having difficulties with the scripts. The serial monitor can be accessed in the Arduino IDE by going to the “Tools” tab and clicking on “Serial Monitor”. I have included parts of the script that can be read in the Arduino serial monitor, just in case you are having difficulties. These sections are flagged by “DEBUGGING” and “END DEBUGGING” in the Arduino code. If you remove the “//” at the beginning of the lines between “DEBUGGING” and “END DEBUGGING” then you can use the serial monitor to read the output from the Arduino. Note that the “SMIDIBT_Read” scripts contain two debugging sections to let you know if the correct command is received, and then the note and velocity bytes. The serial monitor is also useful for receiving errors that might arise while using the SMIDIBT. Note that the baudrate in the serial monitor (in the bottom right corner of the Arduino IDE) has to match the baudrate used in the Arduino code (this should remain at 9600, as defined in the “SETUP” section of the code).

5.3 The MATLAB Scripts Aren’t Working!

That’s not good. Please double-check that you followed the instructions and, perhaps, contact someone who is a MATLAB expert (if you aren’t one already). If that fails (or is not an option), please email me with the error code and MATLAB version: benjamin.glenn.schultz@gmail.com.

5.4 I’m having a problem you haven’t thought of!

Inconceivable! If you do, however, come across a problem or issue that I have not documented here, please let me know. I would gladly try my best to get you up and running. Please email me: benjamin.glenn.schultz@gmail.com with your name, OS, Arduino version, MATLAB version, and a description of your problem.

6. Citing this software

If you use any of the scripts or codes that have been shown in this manual or from the website (www.band-lab/smidib_toolbox/, please cite the associated article:

Schultz, B. G. (2018). The Schultz MIDI Benchmarking toolbox for MIDI interfaces, percussion pads, and sound cards. *Behavior Research Methods.*

6.1 I can't find your reference online!

Just a warning – this paper has not been published (yet) and is, in fact, still being reviewed. In the meantime, I would be happy to send you a draft of the paper until it is accepted.

6.2 Contributions

This project has followed on from a previous collaboration between Benjamin Schultz and Floris van Vugt (Schultz & van Vugt, 2016). I (Benjamin Schultz) have implemented all of the MIDI protocols through the very useful forums available on the Arduino website (www.Arduino.cc), several Instructables pages (www.instructables.com), and some trial and error. I am indebted to Floris and these forums for providing valuable insights into how these systems work and the best way to communicate between different human interface devices.

7. References

Adafruit (2015). Adafruit Wave Shield for Arduino kit, http://www.adafruit.com/product/94

Arduino (2015). http://www.arduino.cc/

GPL v3 (2015), http://www.gnu.org/copyleft/gpl.html

Knörig, A., Wettach, R., & Cohen, J. (2009, February). Fritzing: a tool for advancing electronic prototyping for designers. In Proceedings of the 3rd International Conference on Tangible and Embedded Interaction (pp. 351-358). ACM.

Schultz, B. G. (2018). The Schultz MIDI Benchmarking Toolbox for MIDI interfaces, percussion pads, and sound cards. *Behavior Research Methods, under review.*

Schultz, B. G., & van Vugt, F. T. (2016). Tap Arduino: An Arduino microcontroller for low-latency auditory feedback in sensorimotor synchronization experiments. *Behavior Research Methods*, *48*(4), 1591-1607.

van Vugt, F. T. & Schultz, B. G. (2015). Floris T. van Vugt & Benjamin G. Schultz (2015). Taparduino v1.0. *Zenodo. DOI:* 10.5281/zenodo.16168

Appendix A:
List of components for Arduino

Item	Links
Arduino MEGA or Due	http://store.arduino.cc/
Square force sensitive resistor (FSR 406)	http://www.interlinkelectronics.com/standard-products.php
TRRS 3.5mm Headphone Jack Breakout	https://www.sparkfun.com/products/11570
Resistors<br>10kΩ, brown, black, orange, gold<br>220Ω, red, red, brown, gold<br>470Ω, yellow, violet, brown, gold	https://learn.sparkfun.com/tutorials/resistors/types-of-resistors
Female MIDI Connector	https://www.sparkfun.com/products/9536
Mini Breadboard	https://learn.sparkfun.com/tutorials/how-to-use-a-breadboard
USB 2.0 Type A to Type B (NOT the mini USB connector)	https://www.sparkfun.com/products/512
Optocoupler	https://www.digikey.com/product-detail/en/vishay-semiconductor-opto-division/6N138/751-1263-5-ND/1731496
Diode	https://www.sparkfun.com/products/8588
Hook up wire (kit)<br>Wire colour unimportant	https://www.sparkfun.com/products/124
Screw terminal (3.5mm, 2-pin)	https://www.sparkfun.com/products/8084
Break away headers (Male)	https://www.sparkfun.com/products/116

Appendix B:
Electronics Vendors

Name	Country/Countries	Link
DigiKey	Worldwide	http://www.digikey.com/
Robotshop	U.S.A./Canada/UK/Europe	http://www.robotshop.com
Sparkfun	U.S.A./Canada/UK/Europe/Australia	https://www.sparkfun.com/
JayCar Electronics	Australia	http://www.jaycar.com.au/

Please help us add to this list for your country! Send your country and vendor name and website to benjamin.glenn.schultz@gmail.com or f.t.vanvugt@gmail.com.
